# Clinical and Molecular Characterizations of Carbapenem-Resistant Klebsiella pneumoniae Causing Bloodstream Infection in a Chinese Hospital

**DOI:** 10.1128/spectrum.01690-22

**Published:** 2022-10-03

**Authors:** Na Zhang, Lihua Qi, Xiong Liu, Meiling Jin, Yuan Jin, Xiaojing Yang, Jiali Chen, Shiyu Qin, Fangni Liu, Yue Tang, Ruizhong Jia, Xiushan Zhang, Yong Wang, Jinpeng Guo, Jie Liu, Changjun Wang, Yong Chen

**Affiliations:** a School of Public Health, China Medical University, Shenyang, Liaoning province, People’s Republic of China; b Chinese PLA Center for Disease Control and Prevention, Beijing, China; c Department of Clinical Laboratory, Seventh Medical Center of Chinese PLA General Hospital, Beijing, China; d State Key Laboratory of Pathogen and Biosecurity, Beijing Institute of Biotechnology, Beijing, China; e College of Public Health, Zhengzhou University, Zhengzhou, Henan province, China; Louis Stokes Cleveland VAMC

**Keywords:** bloodstream infection, carbapenem resistance, genomic analysis, genomic evolution, transmission

## Abstract

Bloodstream infection (BSI) caused by carbapenem-resistant Klebsiella pneumoniae (CRKP) is a serious and urgent threat for hospitalized patients. This study aims to describe the clinical and molecular characteristics of CRKP causing BSI in a tertiary-care hospital in Beijing, China. A total of 146 CRKP strains and 39 carbapenem-susceptible K. pneumoniae (CSKP) strains collected in the hospital from 2017 to 2020 were sent for whole-genome sequencing. Univariate and multivariate analyses were used to evaluate risk factors for in-hospital mortality of CRKP-BSI cases. Thirty (20.5%) of 146 CRKP-BSI patients and three (7.7%) of 39 CSKP-BSI patients died at discharge (χ^2^ = 3.471, *P = *0.062). Multivariate logistic regression analysis indicated that age and use of urinary catheters were independent risk factors for the death of CRKP-BSI. The 146 CRKP isolates belonged to 9 sequence types (STs) and 11 serotypes, while the 39 CSKP isolates belonged to 23 STs and 27 serotypes. The mechanism of carbapenem resistance for all the CRKP strains was the acquisition of carbapenemase, mainly KPC-2 (*n* = 127). There were 2 predominant serotypes for ST11 CRKP, namely, KL47 (*n* = 82) and KL64 (*n* = 42). Some virulent genes, including *rmpA2*, *iucABCD* and *iutA*, and *repB* gene, which was involved in plasmid replication, were detected in all ST11-KL64 strains. Evolutionary transmission analysis suggested that ST11 CRKP strains might have evolved from KL47 into KL64 and were accompanied by multiple outbreak events. This study poses an urgent need for enhancing infection control measures in the hospital, especially in the intensive care unit where the patients are at high-risk for acquiring CRKP-BSI.

**IMPORTANCE** CRKP-BSI is demonstrated to cause high mortality. In this study, we demonstrated that ST11 CRKP strains might carry many virulent genes. Meanwhile, outbreak events occurred several times in the strains collected. Carbapenemase acquisition (mainly KPC-2 carbapenemase) was responsible for carbapenem resistance of all the 146 CRKP strains. As 2 predominant strains, all ST11-KL64 strains, but not ST11-KL47 strains, carried *rmpA2*, *iucABCD*, *iutA*, as well as a plasmid replication initiator (*repB*). Our study suggested that the occurrence of region-specific recombination events manifested by the acquisition of some virulence genes might contribute to serotype switching from ST11-KL47 to ST11-KL64. The accumulation of virulent genes in epidemic resistant strains poses a great challenge for the prevention and treatment of BSI caused by K. pneumoniae in high-risk patients.

## INTRODUCTION

Klebsiella pneumoniae, living widely in the host-related environmental ecology, is a commonly conditional pathogenic bacteria in the clinic (1–3). It can continuously acquire new genetic material, produce multi-drug resistance (MDR) and extensive drug resistance (XDR) phenotypes. The widespread of antibiotic-resistant K. pneumoniae has been associated with higher mortality (4, 5), especially carbapenem-resistant K. pneumoniae causing bloodstream infection (CRKP-BSI) (6, 7). The main types of K. pneumoniae infections include respiratory tract infections, urinary tract infections, and BSI (8).

In classic K. pneumoniae, carbapenemase acquisition is a major drug resistance mechanism. The carbapenemase encoding genes are located in bacterial plasmids or chromosomes, which can spread between strains through mobile elements, such as transposons or conjugative plasmids (9). CG258 is the major clone lineage of carbapenem-resistant K. pneumoniae (CRKP), including sequence types (STs) ST270, ST340, ST379, ST407, ST418, ST258, ST11 and other STs. In Europe and America, ST258 is the most common ST of CRKP (10), while in China, the main ST is ST11 (11, 12).

In recent years, the occurrence of nosocomial bloodstream infections caused by CRKP has been increasing. The percentage of CRKP in K. pneumoniae causing BSI has increased to 42% in 2019 in a tertiary hospital in north China (13). It is often accompanied by multiple complications, poor prognosis, and high mortality (14), and needs to be resolved urgently (15). There have been some studies focusing on the epidemiology of bloodstream infection caused by CRKP (16–18). However, the genomic characterization of BSI-related CRKP in different hospitals is still to be elucidated.

To gain a deeper understanding of clinical and molecular characterizations of CRKP-BSI, whole-genome sequencing, molecular, and phenotypic experiments were used to analyze 146 CRKP strains and 39 carbapenem-susceptible K. pneumoniae (CSKP) strains collected from a tertiary-care hospital in Beijing from 2017 to 2020. The transmission and evolution characteristics of CRKP-BSI in this hospital were described.

## RESULTS

### Antimicrobial resistance and hypermucoviscosity phenotype.

A total of 146 CRKP and 39 CSKP strains were included in this study. The antimicrobial susceptibility testing showed that 146 CRKP strains exhibited high resistance to cephalosporins (100%), aminoglycosides (89.7%), and quinolones (89.7%) (Fig. S1). The MIC distribution of 3 carbapenem drugs was shown in Fig. S2, where 125 (85.6%) of 146 CRKP strains exhibited MICs of imipenem ≥8 mg/L, while more than 83.6% of CRKP strains exhibited MICs of meropenem and ertapenem ≥32 mg/L. String test showed that 17 strains belonged to hypermucoviscosity (HMV) strains, including 14 ST11 CRKP strains, 1 ST22 CRKP strain and 2 CSKP strains (ST412 and ST35) (Table S1).

### Clinical characterizations of carbapenem-resistant K. pneumoniae causing bloodstream infection.

The 185 strains were mainly cultured from infants (62.2%, 115/185) and the aged patients ≥60 years old (21.1%, 39/185). The age and gender distribution did not differ significantly between 146 CRKP-BSI patients and 39 CSKP-BSI patients (*P > *0.05). Thirty (20.5%) of 146 CRKP-BSI patients and 3 (7.7%) of 39 CSKP-BSI patients died at discharge, for which the difference was not statistically significant (χ^2^ = 3.471, *P = *0.062). The median age of 30 CRKP-BSI patients died during hospitalization, and 116 CRKP-BSI patients who survived during hospitalization were 61.5 years old (Inter Quartile Range [IQR]: 78 years old) and 47.5 years old (IQR: 53 years old). There were statistically significant differences in intensive care unit (ICU) admission, hospital stay, and use of urinary catheters between the 2 groups (*P < *0.05). A higher proportion of HMV phenotype and a higher number of virulence genes have also been observed among the 30 CRKP-BSI strains, which were cultured from the patients who died during hospitalization (Table 1). Multivariable logistic regression showed that age (OR = 3.51, 95% CI: 2.29-5.97, *P < *0.001) and use of urinary catheters (Odds ratio [OR] = 5.41, 95% confidence interval [CI]: 1.30-36.97, *P = *0.037) were 2 independent risk factors for the CRKP-BSI patients who died at discharge (Table 2). Hosmer-Lemeshow test showed *P = *0.083, suggesting that the logistic regression model was reliable.

**TABLE 1:** Clinical Characteristics of 146 bloodstream infection patients caused by carbapenem-resistant K. pneumoniae

Variables	Clinical outcome during hospitalization no. of patients (%)		
	Death (*n* = 30)	Survive (*n* = 116)	*P*-value
Age (yrs), median (IQR^*a*^)	1(50)	1(53)	0.680
≤18	3(10%)	92(79.3%)	<0.001 0.618 <0.001
19–59	5(16.7%)	13(11.2%)	
≥60	23(76.7%)	10(8.6%)	
Male	19(63.3%)	69(59.5%)	0.701
ICU^*b*^ admission	28(93.3%)	78(67.2%)	0.004
Hospital stay, days, median (IQR)	61.5(78)	47.5(53)	0.543
Underlying diseases			
Cardiovascular disease	1(3.3%)	5(4.3%)	1.000
Chronic lung disease	0	0	—^*e*^
Hematologic malignancy	0	0	—
Diabetes mellitus	0	0	—
Solid tumor	0	0	—
Liver diseases	0	0	—
Renal diseases	1(3.3%)	1(0.9%)	0.875
Premature infant	1 (3.3%)	8(6.9%)	0.766
Other	2(6.7%)	1(0.9%)	0.202
Invasive procedure			
Urinary catheter	29(96.7%)	69(59.5%)	<0.001
CVC^*c*^	13(43.3%)	37(31.9%)	0.239
Ventilation	28(93.3%)	93(80.2%)	0.088
Tracheostomy	6(20.0%)	9(7.8%)	0.103
Blood transfusion	25(83.3%)	91(78.4%)	0.555
Previous surgery	3(10.0%)	116(100.0%)	1.000
Use of antibiotics	30(100.0%)	112(96.6%)	1.000
Cephalosporins	30(100.0%)	116(100.0%)	—
Monocyclic β-Lactam	30(100.0%)	112(96.6%)	0.686
Carbapenems	30(100.0%)	116(100.0%)	—
Aminoglycosides	27(90.0%)	87(75.0%)	0.077
Quinolones	29(96.7%)	102(86.9%)	0.286
Hypermucovisity strains	10(33.3%)	5(4.3%)	<0.001
No. of virulence genes^*d*^ in the strains	26(86.7%)	33(28.4%)	<0.001
1	3(10.0%)	7(6.0%)	0.718
2	2(6.7%)	7(6.0%)	1.000
3	7(23.3%)	6(5.2%)	0.006
4	14(46.7%)	13(11.2%)	<0.001

a IQR, Inter Quartile Range.

b ICU, intensive care unit.

c CVC, central venous catheter.

d Virulence genes include *rmpA*, *rmpA2*, *iucA*, *iroB* and *peg-344*.

e —, *P* value was unavailable.

**TABLE 2:** Multivariate logistic regression analysis of risk factors for CRKP-BSI^*a*^

Variables	Or	95% CI	*P*
Age(yrs)	3.51	2.29-5.97	<0.001
Urinary catheter	5.41	1.3-36.97	0.036

a CRKP, Carbapenem-resistant Klebsiella pneumoniae; BSI, bloodstream infections; OR, Odds ratio; CI, Confidence interval.

### Distribution of multilocus sequence typing types, serotypes, and capsular wzi types.

**FIG 1:**
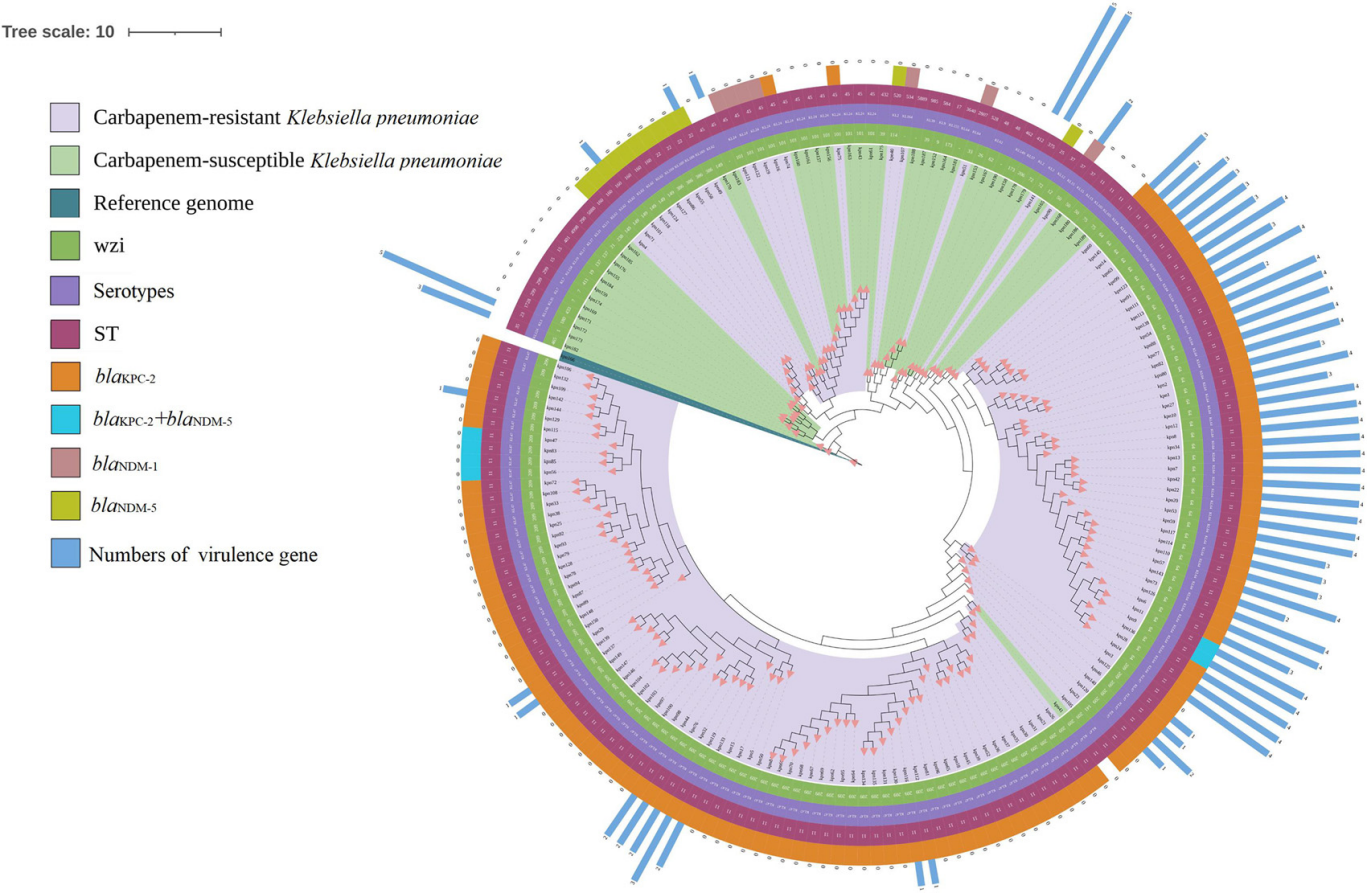
Pan-genome and phylogenetic analyses of 185 Klebsiella pneumoniae. The phylogenetic tree was structured by kSNP3, beautified, and visualized with iTOL. Isolates with light green branches are carbapenem-susceptible K. pneumoniae, and isolates with lavender branches are carbapenem-resistant K. pneumoniae. Virulence genes include *rmpA*, *rmpA2*, *iroB*, *iucA* and *peg-344*. Reference genome about KPN166 (Genome Accession: JAJQMM000000000) was a Klebsiella michiganensis, which was isolated from a tertiary hospital in 2018/7/31, Beijing, China. Empty positions of serotypes represent unknown serotypes and “-” means unknown wzi capsular types.

The whole-genome sequencing assembled information of 185 isolates were shown in Fig. S3, the average of genomic size was 5.62 Mb, and the median of GC % content was 57.2%. All the 185 isolates were identified as *K. pneumonia sensustricto*. The multilocus sequence typing (MLST) types (STs) and serotypes were determined from the WGS data. The 146 CRKP isolates belonged to 9 STs and 11 serotypes (including 2 unknown serotypes), while the 39 CSKP isolates belonged to 23 STs and 27 serotypes (including 5 unkown serotypes). ST11 was the most common ST type in both CRKP isolates (*n* = 125, 85.6%) and CSKP isolates (*n* = 4, 10.3%). KL47 (*n* = 81, 55.5%) and KL64 (*n* = 43, 29.5%) were 2 dominate serotypes in CRKP isolates, while KL24 (*n* = 7, 17.9%) was the most common serotype in CSKP isolates. There were 12 capsular wzi types in CRKP and 26 capsular wzi types in CSKP. The genetic relationship of 185 K. pneumoniae was shown in Fig. 1, which indicated that ST11 CRKP strains mainly corresponded to serotype KL47 and KL64.

### Presence of resistance and virulence genes.

**FIG 2:**
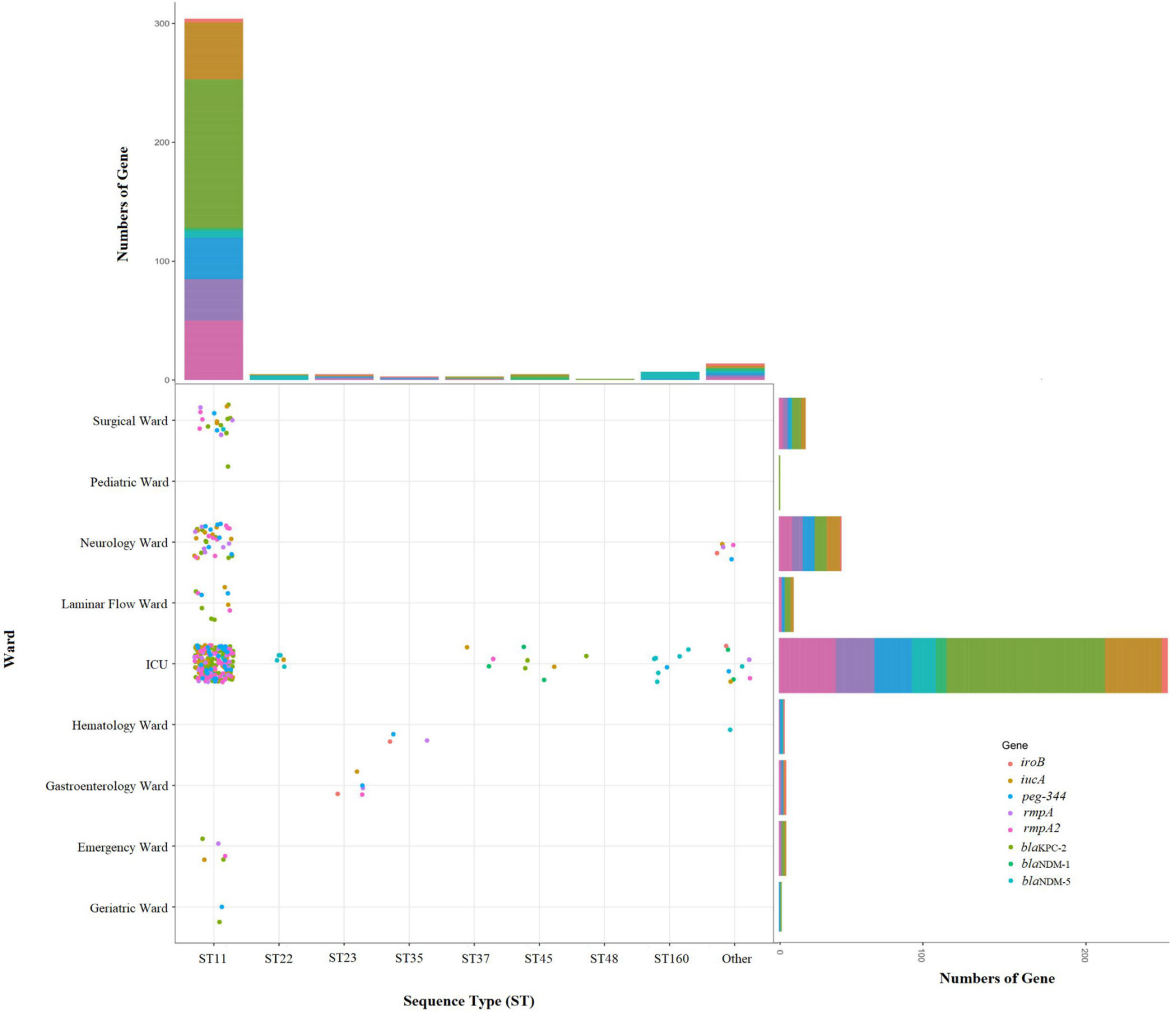
The distribution of department sources, ST types, and presence of virulence and resistance genes in 185 Klebsiella pneumoniae isolates. The solid dots of different colors represent strains that carry different hypervirulent and resistance genes. The cumulative histograms outside the scatterplot indicate the number of genes which belong to different ST types or department sources. ICU, Intensive Care Unit.

All CRKP-BSI strains carry carbapenemase genes, which were dominated by *bla*_KPC-2_, *bla*_NDM-1_, and *bla*_NDM-5_. Of the 146 CRKP isolates, 127 (87.0%) carried the *bla*_KPC-2_ gene, while 19 (13.0%) CRKP isolates carried *bla*_NDM-1_ or *bla*_NDM-5_ gene. The ST11-KL64 CRKP isolates carried at least 2 virulence genes (Fig. 1). A total of 65.6% of K. pneumoniae isolates carrying virulence genes belong to KL64, while 21.8% belong to KL47. The distribution of ST types, major virulence, and resistance genes in strains from different ward sources is shown in Fig. 2.

### Temporal distribution of carbapenemase-producing isolates.

**FIG 3:**
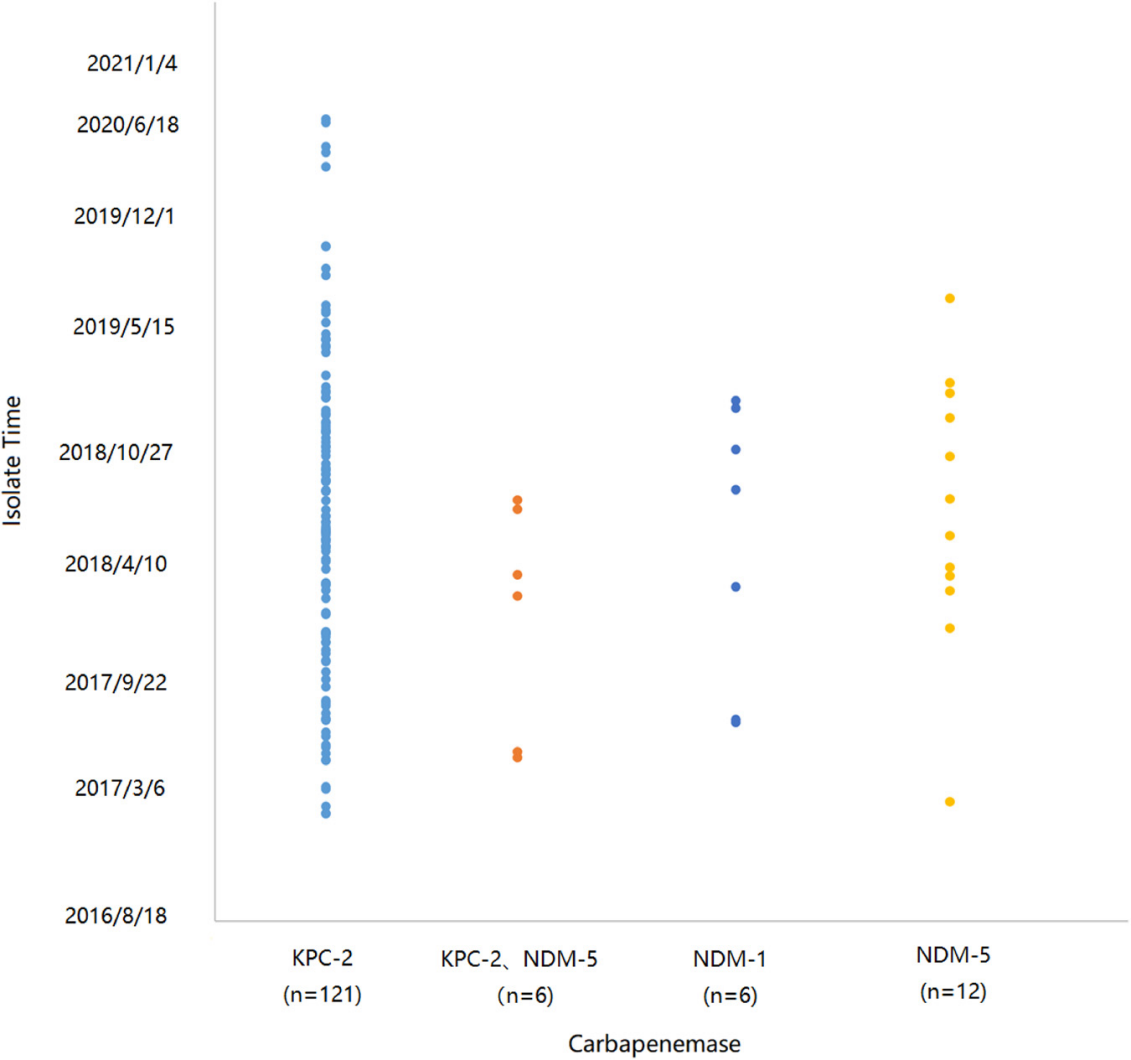
Temporal distribution of 146 Carbapenem-resistant Klebsiella pneumoniae isolates. A spot represents an isolate, blue spots, orange spots, green spots, and yellow spots indicate isolates carrying *bla*_KPC-2_, *bla*_KPC-2_ and *bla*_NDM-5_, *bla*_NDM-1_, *bla*_NDM-5_, respectively. *bla*_KPC-2_ was the mainly collected carbapenem drug resistance gene during 2017–2020. Other carbapenemase genes appeared occasionally.

The 146 CRKP isolates were plotted with strains isolation date to explore the distribution of carbapenem-producing genes (*bla*_KPC-2_, *bla*_NDM-1_, *bla*_NDM-5_) at different periods (Fig. 3). There were 6 ST11 CRKP isolates that carried both *bla*_KPC-2_ and *bla*_NDM-5_ genes, 2 of which were KL64, and 4 were KL47. Among these CRKP-BSI strains, the collection time of each sample was very concentrated, and they mainly carried *bla*_KPC-2_. We speculate that there was clonal transmission among these CRKP strains.

### Several outbreak events occurred in different periods.

**FIG 4:**
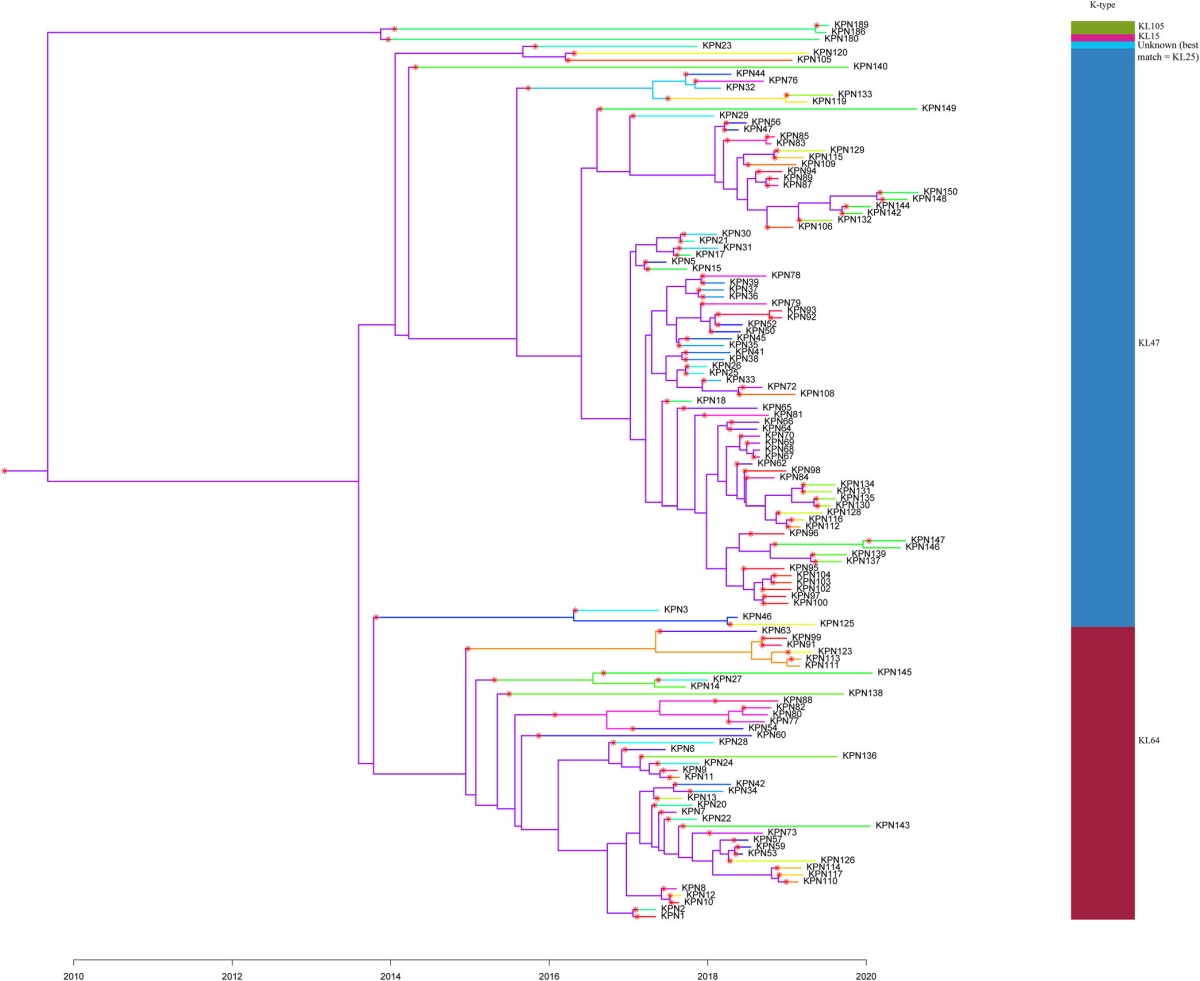
A phylogenetic tree simulating the evolutionary relationships of strains. A transmission event is represented by a change from one color to another, which is highlighted with red stars. The shift in color from left to right indicates that a transmission event occurred between them, which is marked with a red star. The date of the star corresponds to the date of the transmission event, *x* axis represents the isolation time, and the colored tree includes all the information shown in the phylogenetic and transmission trees.

A phylogenetic tree was drawn in combination with the time of sample collection, which can show the evolutionary relationships and outbreak events between the 129 ST11 strains (Fig. 4). We found that the 129 ST11 K. pneumoniae were divided into 2 main evolutionary branches predicted from the single nucleotide polymorphism (SNP) matrix plot (Fig. S4), 1 KL15 and 2 KL105 strains forming a small clade, and the other large clade divided into 2 branches, ST11-KL47 and ST11-KL64. SNP-based phylogenetic tree and Markov chain Monte Carlo (MCMC) procedure was used to infer the evolutionary relationship of transmission between strains, and the ST11-KL47 strain KPN67 (isolated from an infant patient in ICU) was considered as the evolutionary ancestor of 126 isolates, that then evolved into KL25, KL64, and KL47 strains. All ST11-KL64 strains might have evolved from ST11-KL47. Apparently, more than 5 transmission events occurred, including KPN2 to KPN1, KPN21 to KPN30, KPN93 to KPN92, KPN83 to KPN85, and KPN146 to KPN147 (Fig. S5), because of the overlap in ward admission and time of hospitalization stay between every 2 strains.

There were 133 to 279 SNPs that separated ST11-KL47 from ST11-KL64 isolates after the removal of recombination regions. Furthermore, we detected 10 recombination events of >3kb on the branch where strain ST11-KL64 was located, whereas the ST11-KL47 strains did not have recombination events in these regions. Meanwhile, 2 of these 10 recombination events were localized around the *cps* region, which was consistent with a switch from KL47 to KL64 (Fig. S6). Genomic data of 122 ST11-KL64 CRKP from public databases were collected from 2012 to 2020, and 82 ST11-KL47 CRKP strains in this study were used to plot a phylogenetic tree and found that there was no ST11-KL64 CRKP strain isolated at other institutions or in foreign countries that are closely related to ST11-KL47 in this study (Fig. S7).

### All ST11-KL64 strains carried *rmpA2*, *iucABCD*, *iutA*, and *repB* genes.

ST11-KL47 was the most common CRKP strain in this study, but 82.9% (68/82) of them lacked virulence genes. However, all the ST11-KL64 strains carried *rmpA2*, *iucABCD*, and *iutA* genes, which might be co-located on the same plasmid and these were pivotal genes to differentiate ST11-KL64 from ST11-KL47 (Fig. S8). Three *rmpA2*-positive ST11-KL64 isolates also carried *iroB*, and a plasmid-borne virulence factor *peg-344* was exclusively found in 28 of 42 *rmpA2*-positive ST11-KL64 isolates. Fourteen (33.3%) ST11-KL64 isolates showed HMV phenotype, while no ST11-KL47 strains showed HMV phenotype. In addition, *repB*, which is the essential gene for autonomous replication, was found in the plasmid carried by all the ST11-KL64 strains, while it was not found in any ST11-KL47 strains.

Further analysis suggested that the *rmpA2*, *iucABCD*, *iutA*, and *repB* genes in all ST11-KL64 CRKP strains were carried by an IncFIB(K)-IncHI1B-type plasmid with a size of about 217 kb, which showed high identity to a virulence plasmid pVir_020079 (CP029383) detected in Sichuan, China in 2016. The acquisition of *rmpA2*, *iucABCD*, and *iutA* genes might be induced by the gain or loss of gene clusters involved in heavy metal resistance and mobile genetic elements (19).

### Evolution relationship of 115 ST11-KL64 CRKP strains from our study and public database.

To explore the relationships of 42 ST11-KL64 CRKP causing BSI in this study with ST11-KL64 strains from previous studies, we downloaded 73 genome assemblies of ST11-KL64 strains from NCBI database. The information of the 115 ST11-KL64 strains analyzed is listed in Table S2. Twenty-six (56.5%) of 46 ST11-KL64 isolates in our study were clustered in a unique branch that differed from isolates from previous studies. We also found that strains from the same geographical location showed a higher genetic similarity (Fig. S9).

## DISCUSSION

HMV phenotype and biofilm production are key factors for CRKP colonization in the host patients (20, 21). Carbapenem-resistant, HMV K. pneumoniae isolates causing bloodstream infection has been suggested to be associated with high mortality rates (5). We found that some HMV strains didn’t carry virulence genes. For example, KPN86 had HMV phenotype but carried no virulence gene. It suggested that string test alone could not be used to determine the virulence of strains. HMV phenotype was usually used together with the presence of virulence genes to determine hypervirulence in many studies (22–24). KL64 (82.3%, 14/17) was a major capsular locus type of HMV K. pneumoniae and all the HMV KL64 strains belonged to ST11. We also found that there was one ST23-K1 isolate KPN173, which carried 6 virulence genes, including *iucA*, *iroB*, *rmpA*, *rmpA2*, *aerobactin*, and *peg-344*. This isolate had no HMV phenotype but could be regarded as Hvkp (25).

CRKP-BSI is a common comorbidity in ICU (26) with a high-risk factor for mortality (27, 28). There was a high prevalence and clonal spread of CRKP infection in ICU patients in this study, more than 5 transmission events occurred, such as KPN1 and KPN2, which stay in the same ward and have a cross-over time of hospitalization. The phenomenon of numerous strain transmission events and outbreak events in the ICU must be taken seriously. A large number of infant patients were collected in our study, for Neonatology is one of the dominant departments of the hospital that admitted a large number of neonatal patients came from other hospitals every year. It has been suggested that pediatrics was one of the main department sources for CRKP-BSI infection (29), and neonatal sepsis was the leading cause of death (30–32).

The mortality of patients with CRKP-BSI is significantly higher than CSKP-BSI (13), which indicated that carbapenem resistance might contribute to higher mortality for BSI patients. Previous studies found that the risk factors for mortality of CRKP-BSI included long hospitalization, age, insertion of a catheter, bone marrow puncture, use of β-lactamase inhibitor, and noninvasive ventilation (13, 16). Our study also suggested that age and insertion of urinary catheters were 2 independent risk factors for the mortality of CRKP-BSI patients at discharge. Aged patients and infants were the main patient population in our study, and they were vulnerable to CRKP-BSI infection. A total of 81.6% of patients were seriously ill and lived in ICU, while 59.6% of the patients in ICU had a history of urinary catheter use.

Virulence gene acquisition of CRKP is one of the main ways of hypervirulent CRKP (hv-CRKP) production (33). Our study found that 40.4% (59/146) of CRKP strains with bloodstream infection carried virulence genes, while the proportion of classical CRKP strains carrying virulence genes in previous study in China was approximately 34.2% (360/1052) (34), which indicated that CRKP-BSI was more likely to be hv-CRKP. A study (35) proposed that MDR clones were more likely to acquire virulence genes subsequently when compared with hypervirulent clones, while hypervirulent clones are more likely to acquire resistance genes subsequently, which indicated that CRKP-BSI strains that already had resistance genes had advantages to acquire virulence genes subsequently when comparing with CSKP-BSI.

We found that there were 6 CRKP strains carrying both *bla*_NDM-5_ and *bla*_KPC-2_, among which 2 belonged to hv-CRKP and 4 have no pivotal virulence genes (*rmpA*, *rmpA2*, *iucABCD*, *iutA*, and *peg-344*). Sequence blasting found that 5 strains carrying *bla*_KPC-2_ and *bla*_NDM-5_ genes were encoded in the different plasmids, 1 strain named KPN11 carrying *bla*_KPC-2_ was encoded in plasmid and *bla*_NDM-5_ was encoded in the chromosome. Previous studies had reported that one strain carried both *bla*_NDM-5_ and *bla*_KPC-2_ (36), but they were from different plasmids compared with these 6 strains. We guess that there had been abacterial conjugation which leads to the combination of *bla*_KPC-2_ and *bla*_NDM-5_ genes into a plasmid. Another study has demonstrated that the evolution of CRKP is mainly driven by homologous recombination, in contrast to the accumulation of mutations (37), which might help to understand the evolution of CRKP.

By simulating a phylogenetic tree of strains with infection date, we demonstrated that ST11-KL47 is the evolutionary ancestor of ST11-KL64 and identified 35 recombination events in all ST11-KL64 CRKP strains. The *rmpA2*, *iucABCD*, and *iutA* genes were produced during the recombination events. However, we currently have no definitive results to prove that the role of *repB* gene encoded in the plasmid is involved in the conversion of ST11-KL47 to ST11-KL64. Based on the fact that it is a plasmid replication initiator, it is speculated that *repB* may work as a plasmid backbone to facilitate plasmid transfer propagation.

However, our study has some limitations. Firstly, all K. pneumoniae isolates were collected from only one hospital, and the numbers of isolates were not equally distributed in different years, which might have some influence on the accuracy of temporal trends analysis. Secondly, due to the limitation of the retrospective study, we could not obtain the mortality with 30 days after strain isolation for all the patients, so we used clinical outcome at discharge as an alternative indicator. Thirdly, the evolution of ST11 strains was only predicted using BEAST2, and the conclusion might need further laboratory validation. However, our study provided some clues for exploring the mechanisms of CRKP evolution in the future.

In summary, this study demonstrates that there was high mortality (20.5%) for CRKP-BSI patients in the hospital. Multivariate logistic regression analysis indicated that age and the use of urinary catheters were 2 independent risk factors for the death of CRKP-BSI. ST11-KL64 and ST11-KL47 were the 2 most common types of CRKP strains causing BSI in the hospital. The genomic comparison and evolutionary transmission tree analysis suggested that ST11 CRKP might have evolved from KL47 into KL64 in recent years, through the acquisition of some virulence genes encoded in plasmids. This study poses an urgent need for enhancing infection control measures in the hospital to curb the transmission of CRKP isolates.

## MATERIALS AND METHODS

### Isolates source and growth media.

A total of 690 blood samples were sent for bacterial culture during the period of 2017 to 2020 in the hospital, of which 359 strains were K. pneumoniae, including 146 CRKP strains and 213 CSKP strains. All 146 CRKP strains were cultured for WGS. For comparison, 39 CSKP strains that were isolated from human blood in 2017–2020 were randomly selected for further analysis (details are shown in Fig. S10). The 185 strains used in this study were grown at 37°C for 18–24 h on Luria-Bertani (LB) agar plates unless otherwise specified.

### Determination of antibiotic MICs.

Antimicrobial susceptibility of 185 K. pneumoniae strains was tested through the Vitek 2 Compact System in a clinical laboratory of the hospital. MICs of carbapenem drugs (imipenem, meropenem, and ertapenem) were additionally verified through broth microdilution according to the Clinical and Laboratory Standards Institute (M100, 29th edition, 2019) guidelines. Pseudomonas aeruginosa ATCC27853 and Escherichia coli ATCC 25922 were used as control strains for antimicrobial susceptibility testing.

### String test.

We conducted string tests of the 185 K. pneumoniae strains to explore the relationship between hypervirulence and HMV. All strains were grown at 35°C for 18–24 h on Columbia blood agar medium. It was performed with a standard bacteriologic loop to evaluate HMV, and the formation of viscous strings >5 mm in length was considered positive for string test (38).

### DNA isolation, library preparation, and sequencing.

DNA extraction was performed with a QIAamp DNA minikit (#51306 Qiagen). Lyse, bind, wash, elute, as well as other purification and extraction steps were implemented to obtain DNA according to the manufacturer’s instructions. DNA was monitored on 1% agarose gels. DNA concentration and purity were measured using Qubit 4.0 (Thermo Fisher Scientific) and Nanodrop One (Thermo Fisher Scientific) at the same time. All the 185 strains were selected for WGS. Sequencing libraries were generated using NEB Next Ultra DNA Library Prep Kit for Illumina (New England Biolabs) following the manufacturer's recommendations and index codes were added. The library quality was assessed on the Qubit 4.0 Fluorometer (Life Technologies) and Qsep400 High-Throughput Nucleic Acid Protein Analysis System (Houze Biological Technology Co.). At last, DNA libraries were constructed with 150-bp paired-end fragments and WGS was performed using the Illumina Novaseq 6000.

### Assembly and annotation.

Whole-genome sequencing reads were assembled using the Spades software (v3.9.0) into many contigs, retaining those longer than 500 nt as final splicing. According to the identity, e-value, and score values, the comparison results with low reliability were filtered out, and the best result was selected as the result of gene function annotation. Adapter reads resulted in about 0.98 Gb of clean data, and the remaining 185 assembled contigs totaled 5.6 Mb with a GC content of approximately 56.7%. The assembly was annotated by Prokka v1.12 (39).

### Multilocus sequence typing, virulence, resistance gene screening, and phylogenetic analysis.

MLST and capsular locus were determined by analyzing each genome with Kleborate v2.2.0 (40, 41). Unknown MLSTs were verified by stringMLST v0.6.3 (42). Resistance genes, virulence genes, and plasmids were identified and predicted by the screening of contigs in assembled genomes with abricate v0.8. The best reference genome GCF_000240185 was chosen by querying with WGS reads with FastANI v1.33 (43). Seven housekeeping genes of K. pneumoniae on the phylogenetic trees were drawn by kSNP v3.1, visualized by iTOL and R v4.0.2 with the ggtree package v2.2.4. Meanwhile, the heatmap was visualized by TBtools (44).

### Transmission inference.

Variant sites relative to the reference sequence were determined by snippy v3.1, and the optimal model was determined by evaluating and testing in modeltest-ng. A maximum-likelihood phylogenetic tree was inferred from this recombination-free chromosomal alignment using RAxML-NG v1.0.1, implementing the HKY+I+G4 model with 100 bootstrap replicates. Then, ClonalFrameML v1.11 was used to identify and exclude recombination in the resulting alignment results. The SNP sites from the recombination-free chromosomal alignment were extracted by using the snp-sites tool and then beast v2.6.6 was used to infer the transmission time to the most recent common ancestor (tMRCA) (45). The GTR substitution model was selected and allowed for the strict model of the evolutionary rate. MCMC procedure was run for 2,400,000,000 iterations, and convergence of the chain was inspected using Tracer v1.6.0, with each effective sample size (ESS) more than 200 to ensure convergence. The maximum clade credibility (MCC) tree under each model was generated in TreeAnnotator and plotted in Figtree v1.4.4. Transmission tree was drawn by R v4.0.2 with the ggtree (46) and Transphylo (47) package. Transmission matrix visualizing transmission rates between strains were structured by snp-dists v0.8.2 and visualized by R v4.0.2 with the pheatmap package.

### Statistical analysis.

R v4.0.2 was used to perform all the statistical analysis. Continuous variables were described as median and IQR, which was compared using the Mann-Whitney U test. Potentially dangerous variables were included in the multivariable logistic regression model. We calculated the ORs and the 95% CIs for each variable. *P < *0.05 were considered statistically significant. The Hosmer-Lemeshow test was used to test the goodness of fit for the logistic regression model, and the logistic model is reliable when *P > *0.05 in this test.

### Ethics statement.

The institutional ethics committees of The Seven Medical Center of PLA General Hospital, Beijing, China approved the study (2021-82). As all data were anonymously collected and interpreted, the institutional ethics committees waived the need for written informed consent from the participants.

### Data availability.

All complete genome sequences are available in GenBank, and organized under BioProject PRJNA785065 (details are shown in Table S3).
