# Supplementary material for: Clinical and Molecular Characterizations of Carbapenem-Resistant Klebsiella pneumoniae Causing Bloodstream Infection in a Chinese Hospital
Source: Microbiol Spectr. 2022 Oct 3;10(5):e01690-22. doi: 10.1128/spectrum.01690-22 (PMC9603270; doi:10.1128/spectrum.01690-22)
Supplement: Supplemental file 1 — Fig. S1-S10; Tables S1-S5. Download spectrum.01690-22-s0001.pdf, PDF file, 0.9 MB [file spectrum.01690-22-s0001.pdf]

Clinical and Molecular Characterizations of Carbapenem-resistant *Klebsiella pneumoniae* Causing Bloodstream Infection in a Chinese Hospital

Supplementary document

Tables

Table S1 MLST, serotype, resistance genes and virulence genes of hypermucoviscous isolates.

Strain Name	ST	Serotype	*bla*$_{KPC-2}$	*bla*$_{NDM-1}$	*bla*$_{NDM-5}$	*iucA*	*iroB*	*rmpA*	*rmpA2*	*peg-344*
KPN8	11	KL64	+	-	-	+	-	+	+	+
KPN9	11	KL64	+	-	+	+	-	+	+	+
KPN24	11	KL64	+	-	-	+	-	+	+	+
KPN28	11	KL64	+	-	-	+	-	+	+	+
KPN53	11	KL64	+	-	-	+	-	+	+	+
KPN57	11	KL64	+	-	-	+	-	+	+	+
KPN59	11	KL64	+	-	-	+	-	+	+	+
KPN77	11	KL64	+	-	-	+	-	+	+	+
KPN86	22	KL169	-	-	+	-	-	-	-	-
KPN91	11	KL64	+	-	-	+	-	+	+	+
KPN99	11	KL64	+	-	-	+	-	+	+	-
KPN111	11	KL64	+	-	-	+	-	+	+	-
KPN117	11	KL64	+	-	-	+	-	+	+	-
KPN123	11	KL64	+	-	-	+	-	+	+	-
KPN136	11	KL64	+	-	-	+	-	+	+	+
KPN178	412	KL57	-	-	-	+	+	+	+	+
KPN182	35	KL124	-	-	-	-	+	+	-	+

“-” indicates negative of gene detection and “+” indicates positive of gene detection.

Table S2 Genomic information of 115 ST11-KL64 *Klebsiella pneumonia*e isolates.

Strain Name	Assembly	Species	Isolate time	K_serotype	ST	N50	Genome_Size(bp)
GCF002164835	GCF_002164835.3	*Klebsiella pneumoniae*	2018/6/12	KL64	ST11	5476966	5670138
GCF001887985	GCF_001887985.3	*Klebsiella pneumoniae*	2018/3/19	KL64	ST11	5461116	5632871
GCF002587165	GCF_002587165.1	*Klebsiella pneumoniae*	2017/10/18	KL64	ST11	5484708	5880984
GCF002811325	GCF_002811325.2	*Klebsiella pneumoniae*	2018/4/17	KL64	ST11	5444817	5641106
GCF002164855	GCF_002164855.2	*Klebsiella pneumoniae*	2018/8/27	KL64	ST11	5421726	5610557
GCF002852975	GCF_002852975.2	*Klebsiella pneumoniae*	2018/5/21	KL64	ST11	5468182	5896594
GCF003402995	GCF_003402995.1	*Klebsiella pneumoniae*	2018/8/18	KL64	ST11	5406566	5879323
GCF003112125	GCF_003112125.1	*Klebsiella pneumoniae*	2018/5/10	KL64	ST11	5339740	5945288
GCF900516455	GCF_900516455.1	*Klebsiella pneumoniae*	2018/8/22	KL64	ST11	214375	5657472
GCF001701895	GCF_001701895.1	*Klebsiella pneumoniae*	2016/8/9	KL64	ST11	270649	5860872
GCF001856585	GCF_001856585.1	*Klebsiella pneumoniae*	2016/10/29	KL64	ST11	198147	5727930
GCF002264365	GCF_002264365.1	*Klebsiella pneumoniae*	2017/8/22	KL64	ST11	203939	5525783
GCF002261625	GCF_002261625.1	*Klebsiella pneumoniae*	2017/8/21	KL64	ST11	207426	5487690
GCF002264465	GCF_002264465.1	*Klebsiella pneumoniae*	2017/8/22	KL64	ST11	203896	5581788
GCF003321155	GCF_003321155.1	*Klebsiella pneumoniae*	2018/7/15	KL64	ST11	262972	5839058
GCF002262345	GCF_002262345.1	*Klebsiella pneumoniae*	2017/8/22	KL64	ST11	203939	5535938
GCF002262795	GCF_002262795.1	*Klebsiella pneumoniae*	2017/8/22	KL64	ST11	176166	5607211
GCF003037475	GCF_003037475.1	*Klebsiella pneumoniae*	2018/4/5	KL64	ST11	203335	5515691

GCF003037585	GCF_003037585.1	*Klebsiella pneumoniae*	2018/4/5	KL64	ST11	203335	5515628
GCF002844905	GCF_002844905.1	*Klebsiella pneumoniae*	2017/12/19	KL64	ST11	198350	5585355
GCF003036925	GCF_003036925.1	*Klebsiella pneumoniae*	2018/4/5	KL64	ST11	203335	5550723
GCF003038235	GCF_003038235.1	*Klebsiella pneumoniae*	2018/4/5	KL64	ST11	203335	5515274
GCF003261855	GCF_003261855.1	*Klebsiella pneumoniae*	2018/6/25	KL64	ST11	203078	5468654
GCF003037055	GCF_003037055.1	*Klebsiella pneumoniae*	2018/4/5	KL64	ST11	203874	5512353
GCF003036805	GCF_003036805.1	*Klebsiella pneumoniae*	2018/4/5	KL64	ST11	203335	5646263
GCF002262745	GCF_002262745.1	*Klebsiella pneumoniae*	2017/8/22	KL64	ST11	185958	5548343
GCF002264545	GCF_002264545.1	*Klebsiella pneumoniae*	2017/8/22	KL64	ST11	192313	5733347
GCF003036725	GCF_003036725.1	*Klebsiella pneumoniae*	2018/4/5	KL64	ST11	176106	5686783
GCF003036815	GCF_003036815.1	*Klebsiella pneumoniae*	2018/4/5	KL64	ST11	193036	5673911
GCF003036895	GCF_003036895.1	*Klebsiella pneumoniae*	2018/4/5	KL64	ST11	193036	5735497
GCF002262295	GCF_002262295.1	*Klebsiella pneumoniae*	2017/8/22	KL64	ST11	185047	5623378
GCF003262505	GCF_003262505.1	*Klebsiella pneumoniae*	2018/6/25	KL64	ST11	185639	5443844
GCF003262625	GCF_003262625.1	*Klebsiella pneumoniae*	2018/6/25	KL64	ST11	185639	5573990
GCF003262035	GCF_003262035.1	*Klebsiella pneumoniae*	2018/6/25	KL64	ST11	170244	5510551
GCF002850635	GCF_002850635.1	*Klebsiella pneumoniae*	2018/1/3	KL64	ST11	192840	5918798
GCF003321215	GCF_003321215.1	*Klebsiella pneumoniae*	2018/7/15	KL64	ST11	178889	5870119
GCF002262375	GCF_002262375.1	*Klebsiella pneumoniae*	2017/8/22	KL64	ST11	176166	5804683
GCF002262555	GCF_002262555.1	*Klebsiella pneumoniae*	2017/8/22	KL64	ST11	176058	5727547
GCF003262615	GCF_003262615.1	*Klebsiella pneumoniae*	2018/6/25	KL64	ST11	185639	5585797

GCF003037485	GCF_003037485.1	*Klebsiella pneumoniae*	2018/4/5	KL64	ST11	203677	5723062
GCF002261665	GCF_002261665.1	*Klebsiella pneumoniae*	2017/8/21	KL64	ST11	171002	5684066
GCF003036465	GCF_003036465.1	*Klebsiella pneumoniae*	2018/4/5	KL64	ST11	192210	5724693
GCF002262185	GCF_002262185.1	*Klebsiella pneumoniae*	2017/8/21	KL64	ST11	176166	5775211
GCF002262545	GCF_002262545.1	*Klebsiella pneumoniae*	2017/8/22	KL64	ST11	176058	5746717
GCF002845285	GCF_002845285.1	*Klebsiella pneumoniae*	2017/12/19	KL64	ST11	141255	5579548
GCF003263205	GCF_003263205.1	*Klebsiella pneumoniae*	2018/6/25	KL64	ST11	175912	5712323
GCF002845185	GCF_002845185.1	*Klebsiella pneumoniae*	2017/12/19	KL64	ST11	151791	5818316
GCF002852675	GCF_002852675.1	*Klebsiella pneumoniae*	2018/1/3	KL64	ST11	129701	5580462
GCF002261875	GCF_002261875.1	*Klebsiella pneumoniae*	2017/8/21	KL64	ST11	171861	5793053
GCF002852645	GCF_002852645.1	*Klebsiella pneumoniae*	2018/1/3	KL64	ST11	175954	5747310
GCF003262655	GCF_003262655.1	*Klebsiella pneumoniae*	2018/6/25	KL64	ST11	175912	5724437
GCF003263195	GCF_003263195.1	*Klebsiella pneumoniae*	2018/6/25	KL64	ST11	175912	5740621
GCF002262135	GCF_002262135.1	*Klebsiella pneumoniae*	2017/8/21	KL64	ST11	171958	5710180
GCF002262225	GCF_002262225.1	*Klebsiella pneumoniae*	2017/8/22	KL64	ST11	142391	5807952
GCF003036785	GCF_003036785.1	*Klebsiella pneumoniae*	2018/4/5	KL64	ST11	175912	5728625
GCF003262535	GCF_003262535.1	*Klebsiella pneumoniae*	2018/6/25	KL64	ST11	175912	5760926
GCF002845145	GCF_002845145.1	*Klebsiella pneumoniae*	2017/12/19	KL64	ST11	176066	5772074
GCF002851975	GCF_002851975.1	*Klebsiella pneumoniae*	2018/1/3	KL64	ST11	151791	5762774
GCF002853235	GCF_002853235.1	*Klebsiella pneumoniae*	2018/1/3	KL64	ST11	118036	5779623
GCF003263225	GCF_003263225.1	*Klebsiella pneumoniae*	2018/6/25	KL64	ST11	142762	5613441

GCF003263135	GCF_003263135.1	*Klebsiella pneumoniae*	2018/6/25	KL64	ST11	175912	5933910
GCF002740615	GCF_002740615.1	*Klebsiella pneumoniae*	2017/11/1	KL64	ST11	110558	5774799
GCF003261715	GCF_003261715.1	*Klebsiella pneumoniae*	2018/6/25	KL64	ST11	109905	5426011
GCF003262105	GCF_003262105.1	*Klebsiella pneumoniae*	2018/6/25	KL64	ST11	129253	5542170
GCF002262805	GCF_002262805.1	*Klebsiella pneumoniae*	2017/8/22	KL64	ST11	108899	5744876
GCF003261655	GCF_003261655.1	*Klebsiella pneumoniae*	2018/6/25	KL64	ST11	102153	5499771
GCF003312125	GCF_003312125.1	*Klebsiella pneumoniae*	2018/7/10	KL64	ST11	116850	5714599
GCF002890815	GCF_002890815.1	*Klebsiella pneumoniae*	2018/1/17	KL64	ST11	99389	5820828
GCF002265565	GCF_002265565.1	*Klebsiella pneumoniae*	2017/8/22	KL64	ST11	105849	5828964
GCF002265605	GCF_002265605.1	*Klebsiella pneumoniae*	2017/8/22	KL64	ST11	97699	5828572
GCF003312135	GCF_003312135.1	*Klebsiella pneumoniae*	2018/7/10	KL64	ST11	106089	5850963
GCF003312185	GCF_003312185.1	*Klebsiella pneumoniae*	2018/7/10	KL64	ST11	100529	5944669
GCF003321195	GCF_003321195.1	*Klebsiella pneumoniae*	2018/7/15	KL64	ST11	99738	5848531
kpn1	GCA_021521275.1	*Klebsiella pneumoniae*	2017/2/21	KL64	ST11	192253	5750874
kpn2	GCA_021521235.1	*Klebsiella pneumoniae*	2017/2/22	KL64	ST11	181994	5677994
kpn6	GCA_021521175.1	*Klebsiella pneumoniae*	2017/4/5	KL64	ST11	183749	5943786
kpn7	GCA_021521135.1	*Klebsiella pneumoniae*	2017/5/25	KL64	ST11	176147	5876330
kpn8	GCA_021521145.1	*Klebsiella pneumoniae*	2017/5/26	KL64	ST11	176147	5878775
kpn9	GCA_021521105.1	*Klebsiella pneumoniae*	2017/5/30	KL64	ST11	183749	5996916
kpn10	GCA_021521075.1	*Klebsiella pneumoniae*	2017/6/6	KL64	ST11	192253	5792531
kpn11	GCA_021521095.1	*Klebsiella pneumoniae*	2017/6/9	KL64	ST11	183749	5995066

kpn12	GCA_021521045.1	*Klebsiella pneumoniae*	2017/6/17	KL64	ST11	192253	5791778
kpn13	GCA_021521035.1	*Klebsiella pneumoniae*	2017/6/21	KL64	ST11	176147	5876886
kpn14	GCA_021520995.1	*Klebsiella pneumoniae*	2017/7/6	KL64	ST11	176147	5675935
kpn20	GCA_021520875.1	*Klebsiella pneumoniae*	2017/8/6	KL64	ST11	176149	5814038
kpn22	GCA_021520845.1	*Klebsiella pneumoniae*	2017/8/28	KL64	ST11	142287	5790456
kpn24	GCA_021519715.1	*Klebsiella pneumoniae*	2017/9/6	KL64	ST11	183746	5897356
kpn27	GCA_021520775.1	*Klebsiella pneumoniae*	2017/10/26	KL64	ST11	176147	5717695
kpn28	GCA_021519635.1	*Klebsiella pneumoniae*	2017/11/13	KL64	ST11	183749	5881739
kpn34	GCA_021519515.1	*Klebsiella pneumoniae*	2017/12/26	KL64	ST11	161803	5787658
kpn42	GCA_021519355.1	*Klebsiella pneumoniae*	2018/3/27	KL64	ST11	192253	5757230
kpn53	GCA_021520545.1	*Klebsiella pneumoniae*	2018/3/30	KL64	ST11	175739	5916975
kpn54	GCA_021520475.1	*Klebsiella pneumoniae*	2018/3/29	KL64	ST11	185895	5735583
kpn57	GCA_021518905.1	*Klebsiella pneumoniae*	2018/4/23	KL64	ST11	192253	5906317
kpn59	GCA_021518875.1	*Klebsiella pneumoniae*	2018/5/6	KL64	ST11	175739	5897368
kpn60	GCA_021518815.1	*Klebsiella pneumoniae*	2018/5/24	KL64	ST11	192249	5782004
kpn63	GCA_021518715.1	*Klebsiella pneumoniae*	2018/5/30	KL64	ST11	160372	5773456
kpn73	GCA_021518375.1	*Klebsiella pneumoniae*	2018/6/26	KL64	ST11	176149	5921010
kpn77	GCA_021518345.1	*Klebsiella pneumoniae*	2018/7/6	KL64	ST11	137240	5806987
kpn80	GCA_021518215.1	*Klebsiella pneumoniae*	2018/7/24	KL64	ST11	176147	5808235
kpn82	GCA_021518145.1	*Klebsiella pneumoniae*	2018/8/4	KL64	ST11	176147	5806450
kpn88	GCA_021517915.1	*Klebsiella pneumoniae*	2018/9/6	KL64	ST11	151824	5750417

kpn91	GCA_021517775.1	*Klebsiella pneumoniae*	2018/9/22	KL64	ST11	192297	5796312
kpn99	GCA_021517645.1	*Klebsiella pneumoniae*	2018/10/16	KL64	ST11	166244	5791310
kpn110	GCA_021517215.1	*Klebsiella pneumoniae*	2018/12/7	KL64	ST11	142287	5896407
kpn111	GCA_021517255.1	*Klebsiella pneumoniae*	2018/12/18	KL64	ST11	192297	5793915
kpn113	GCA_021517175.1	*Klebsiella pneumoniae*	2018/12/19	KL64	ST11	166244	5783969
kpn114	GCA_021517115.1	*Klebsiella pneumoniae*	2018/12/23	KL64	ST11	176149	5897581
kpn117	GCA_021516895.1	*Klebsiella pneumoniae*	2019/1/2	KL64	ST11	142287	5896070
kpn123	GCA_021516875.1	*Klebsiella pneumoniae*	2019/2/15	KL64	ST11	216018	5827882
kpn126	GCA_021520015.1	*Klebsiella pneumoniae*	2019/2/27	KL64	ST11	183746	5893168
kpn136	GCA_021519915.1	*Klebsiella pneumoniae*	2019/6/6	KL64	ST11	183749	5917592
kpn138	GCA_021516625.1	*Klebsiella pneumoniae*	2019/7/13	KL64	ST11	192253	5775060
kpn143	GCA_021516415.1	*Klebsiella pneumoniae*	2019/11/5	KL64	ST11	176149	5899322
kpn145	GCA_021516345.1	*Klebsiella pneumoniae*	2019/9/16	KL64	ST11	128977	5782409

Table S3 List of 185 *Klebsiella pneumoniae* isolates used in this study.

Strain Name	Genome Accession	ST	Isolate Source	RawReads	CleanReads	Clean_GC(%)
KPN1	JAJOCB000000000	11	Blood	37571180	35538404	56
KPN2	JAJOCA000000000	11	Blood	46502518	44150266	56
KPN3	JAJOBZ000000000	11	Blood	35757144	33850456	57
KPN4	JAJOBY000000000	160	Blood	33969810	31844282	57
KPN5	JAJOBX000000000	11	Blood	47327012	45023976	57
KPN6	JAJOBW000000000	11	Blood	65232544	62616622	56
KPN7	JAJOBV000000000	11	Blood	37493544	35547238	57
KPN8	JAJOBU000000000	11	Blood	35294404	33275870	56.5
KPN9	JAJOBT000000000	11	Blood	39044130	36964188	56
KPN10	JAJOBS000000000	11	Blood	40193490	38195300	57
KPN11	JAJOBR000000000	11	Blood	50393034	47830418	56
KPN12	JAJOBQ000000000	11	Blood	44971822	42686136	57
KPN13	JAJOBP000000000	11	Blood	41867712	40387628	57
KPN14	JAJOBO000000000	11	Blood	40793128	39388866	57
KPN15	JAJOBN000000000	11	Blood	43722934	41671490	49
KPN16	JAJOBM000000000	45	Blood	43757874	41611750	57
KPN17	JAJOBL000000000	11	Blood	45253846	43103462	57
KPN18	JAJOBK000000000	11	Blood	60531846	58203100	57

KPN19	JAJOBJ000000000	45	Blood	55018556	52942298	56
KPN20	JAJOBI000000000	11	Blood	48663586	46711560	56
KPN21	JAJOBH000000000	11	Blood	54205948	52246700	56
KPN22	JAJOBG000000000	11	Blood	35654832	34447246	57
KPN23	JAJOBF000000000	11	Blood	38166914	36690984	57
KPN24	JAJOBE000000000	11	Blood	41701664	40169166	56
KPN25	JAJOBD000000000	11	Blood	35997764	34790806	57
KPN26	JAJOBC000000000	11	Blood	40928474	39406344	57
KPN27	JAJOBB000000000	11	Blood	33582932	32098318	57
KPN28	JAJOBA000000000	11	Blood	60174230	57865816	56
KPN29	JAJOAZ000000000	11	Blood	50306634	48367762	56
KPN30	JAJOAY000000000	11	Blood	49135808	47190946	57
KPN31	JAJOAX000000000	11	Blood	57583724	55323028	57
KPN32	JAJOAW000000000	11	Blood	56313812	54577400	57
KPN33	JAJOAV000000000	11	Blood	54133388	52135758	56
KPN34	JAJOAU000000000	11	Blood	61171124	59088974	56
KPN35	JAJOAT000000000	11	Blood	58944486	56923302	56
KPN36	JAJOAS000000000	11	Blood	63057330	61051872	56
KPN37	JAJOAR000000000	11	Blood	55362926	53637576	57
KPN38	JAJOAQ000000000	11	Blood	54391640	52601180	56
KPN39	JAJOAP000000000	11	Blood	53750174	51868472	57

KPN40	JAJOAO000000000	520	Blood	33771978	32437596	57
KPN41	JAJOAN000000000	11	Blood	82994768	80055698	56
KPN42	JAJOAM000000000	11	Blood	51940746	50254282	56
KPN43	JAJOAL000000000	45	Blood	56744976	54852358	56
KPN44	JAJOAK000000000	11	Blood	44327530	43010670	57
KPN45	JAJOAJ000000000	11	Blood	47655826	46185038	57
KPN46	JAJOAI000000000	11	Blood	47905576	45708644	57
KPN47	JAJOAH000000000	11	Blood	45995620	43761474	56
KPN49	JAJOAG000000000	22	Blood	40208386	38140222	56.5
KPN50	JAJOAF000000000	11	Blood	40496988	38697196	57
KPN51	JAJQML000000000	2807	Blood	51152566	48622650	57
KPN52	JAJOAE000000000	11	Blood	39620234	37620244	57
KPN53	JAJOCD000000000	11	Blood	37331662	35496100	56
KPN54	JAJOCC000000000	11	Blood	44145918	42521770	56
KPN55	JAJOAD000000000	22	Blood	45917890	43915252	56
KPN56	JAJOAC000000000	11	Blood	42321808	40232308	57
KPN57	JAJOAB000000000	11	Blood	43374020	41180414	57
KPN58	JAJOAA000000000	22	Blood	46945768	44653544	56
KPN59	JAJNZZ000000000	11	Blood	49669264	47196528	56
KPN60	JAJNZY000000000	11	Blood	83188998	80301268	57
KPN61	JAJNZX000000000	45	Blood	64797384	63064432	57

KPN62	JAJNZW000000000	11	Blood	56861820	54126522	57
KPN63	JAJNZV000000000	11	Blood	49906300	47720024	57
KPN64	JAJNZU000000000	11	Blood	31515306	30307296	57
KPN65	JAJNZT000000000	11	Blood	37777440	36409338	57
KPN66	JAJNZS000000000	11	Blood	39283694	38062444	57
KPN67	JAJNZR000000000	11	Blood	34032696	32945486	57
KPN68	JAJNZQ000000000	11	Blood	36919740	35437542	57
KPN69	JAJQMK000000000	11	Blood	40168208	38909090	57
KPN70	JAJNZP000000000	11	Blood	41531538	40113678	57
KPN71	JAJNZO000000000	160	Blood	37110340	35766092	57
KPN72	JAJNZN000000000	11	Blood	45176644	43500808	57
KPN73	JAJNZM000000000	11	Blood	34821240	33590134	56
KPN74	JAJNZL000000000	45	Blood	35592420	34421202	57
KPN75	JAJQMJ000000000	45	Blood	84217836	81524952	57
KPN76	JAJNZK000000000	11	Blood	71443206	69330870	57
KPN77	JAJNZJ000000000	11	Blood	49828236	48087708	57
KPN78	JAJNZI000000000	11	Blood	85894272	83472088	57
KPN79	JAJNZH000000000	11	Blood	42670356	40826098	57
KPN80	JAJNZG000000000	11	Blood	40892162	39677672	57
KPN81	JAJNZF000000000	11	Blood	51773326	50093680	57
KPN82	JAJNZE000000000	11	Blood	44636900	42981898	57

KPN83	JAJNZD000000000	11	Blood	37339484	36031668	57
KPN84	JAJNZC000000000	11	Blood	42689718	41249060	57
KPN85	JAJNZB000000000	11	Blood	35972476	34785988	57
KPN86	JAJNZA000000000	22	Blood	45971992	44266150	56
KPN87	JAJNYZ000000000	11	Blood	43189860	41427202	57
KPN88	JAJNYY000000000	11	Blood	36292906	34862064	57
KPN89	JAJNYX000000000	11	Blood	38956878	37535024	57
KPN90	JAJNYW000000000	37	Blood	38326416	36964618	57
KPN91	JAJNYV000000000	11	Blood	40844836	39385210	57
KPN92	JAJNYU000000000	11	Blood	38084488	36514282	57
KPN93	JAJNYT000000000	11	Blood	42402910	40715304	57
KPN94	JAJNYS000000000	11	Blood	44940826	43187778	57
KPN95	JAJNYR000000000	11	Blood	51972908	49198646	57
KPN96	JAJNYQ000000000	11	Blood	68701436	66817140	57
KPN97	JAJQMI000000000	11	Blood	68642514	66699624	57
KPN98	JAJNYP000000000	11	Blood	62578008	60770008	56
KPN99	JAJNYO000000000	11	Blood	63074884	61099132	57
KPN100	JAJNYN000000000	11	Blood	56247062	54787112	57
KPN101	JAJNYM000000000	160	Blood	71119982	69092268	57
KPN102	JAJQMH000000000	11	Blood	81782596	78494966	57
KPN103	JAJNYL000000000	11	Blood	86858738	83668294	57

KPN104	JAJNYK000000000	11	Blood	89053618	85835420	57
KPN105	JAJNYJ000000000	11	Blood	46046684	43801120	57
KPN106	JAJQMG000000000	11	Blood	79758262	77051506	57
KPN107	JAJQMF000000000	534	Blood	51011304	48363204	57
KPN108	JAJNYI000000000	11	Blood	50175464	47732110	57
KPN109	JAJNYH000000000	11	Blood	46074118	43853758	56
KPN110	JAJNYG000000000	11	Blood	52059026	49970358	56
KPN111	JAJNYF000000000	11	Blood	35186208	33781654	56.5
KPN112	JAJNYE000000000	11	Blood	83278430	80266800	57
KPN113	JAJNYD000000000	11	Blood	75698870	72861900	57
KPN114	JAJNYC000000000	11	Blood	68013166	65727792	56.5
KPN115	JAJNYB000000000	11	Blood	71272842	68714514	57
KPN116	JAJNYA000000000	11	Blood	73060798	70606976	57
KPN117	JAJNXZ000000000	11	Blood	54135928	51707216	56.5
KPN118	JAJQME000000000	160	Blood	69972500	67430470	57
KPN119	JAJNXY000000000	11	Blood	61180920	59007056	57
KPN120	JAJQMD000000000	11	Blood	70562168	67782470	57
KPN121	JAJQMC000000000	45	Blood	78323876	75681848	57
KPN122	JAJQMB000000000	45	Blood	67731214	65466586	57
KPN123	JAJNXX000000000	11	Blood	41389098	39886764	56.5
KPN124	JAJNXW000000000	160	Blood	52542896	50352186	57

KPN125	JAJQMA000000000	11	Blood	61207566	58983344	57
KPN126	JAJQLZ000000000	11	Blood	57529502	54997104	57
KPN127	JAJQLY000000000	160	Blood	63192714	60861282	57
KPN128	JAJNXV000000000	11	Blood	67990210	65743844	57
KPN129	JAJNXU000000000	11	Blood	47431378	45130752	57
KPN130	JAJNXT000000000	11	Blood	47466544	44859850	57
KPN131	JAJNXS000000000	11	Blood	51951578	49225574	57
KPN132	JAJNXR000000000	11	Blood	58357886	56331620	57
KPN133	JAJNXQ000000000	11	Blood	62227458	59931684	57
KPN134	JAJQLX000000000	11	Blood	49200044	46671832	57
KPN135	JAJQLW000000000	11	Blood	60815714	58442116	57
KPN136	JAJQLV000000000	11	Blood	63412980	61063536	56
KPN137	JAJNXP000000000	11	Blood	35726776	34082912	57
KPN138	JAJNXO000000000	11	Blood	64674686	62360510	57
KPN139	JAJNXN000000000	11	Blood	51073252	48452944	57
KPN140	JAJNXM000000000	11	Blood	48811784	46281330	57
KPN141	JAJNXL000000000	25	Blood	49954308	48460866	57
KPN142	JAJNXK000000000	11	Blood	52802376	51362282	57
KPN143	JAJNXJ000000000	11	Blood	51326092	49876872	57
KPN144	JAJNXI000000000	11	Blood	55222514	53702352	57
KPN145	JAJNXH000000000	11	Blood	48636264	47191708	57

KPN146	JAJNXG000000000	11	Blood	51326862	49904108	57
KPN147	JAJNXF000000000	11	Blood	51314508	49781742	57
KPN148	JAJNXE000000000	11	Blood	52167356	49264888	57
KPN149	JAJNXD000000000	11	Blood	53845122	52308180	57
KPN150	JAJNXC000000000	11	Blood	62584728	59766376	57
KPN152	JAJNXB000000000	584	Blood	40434754	38627266	57
KPN153	JAJNXA000000000	528	Blood	73578492	70830118	56
KPN155	JAJNWZ000000000	401	Blood	65005382	62628202	56
KPN156	JAJNWY000000000	45	Blood	64577140	62310396	57
KPN157	JAJNWX000000000	45	Blood	60341956	58085758	57
KPN158	JAJQLU000000000	462	Blood	69231630	66584964	56
KPN159	JAJQLT000000000	15	Blood	58664104	56473610	57
KPN160	JAJNWW000000000	45	Blood	51725584	49556592	57
KPN161	JAJNWV000000000	45	Blood	35214816	33636578	56.5
KPN162	JAJNWU000000000	5888	Blood	49436650	47254862	57
KPN163	JAJNWT000000000	45	Blood	44532350	42534894	57
KPN164	JAJNWS000000000	17	Blood	62103812	59227994	57
KPN165	JAJNWR000000000	37	Blood	58539474	56112894	57
KPN167	JAJNWQ000000000	48	Blood	57087796	54404638	57
KPN168	JAJNWP000000000	37	Blood	43380420	41356878	57
KPN169	JAJNWO000000000	299	Blood	46100050	43872260	55

KPN170	JAJSOP000000000	45	Blood	40077888	38320500	57
KPN171	JAJNWN000000000	299	Blood	45638220	43397774	57
KPN172	JAJQLS000000000	1728	Blood	47139222	44984132	57
KPN173	JAJNWM000000000	23	Blood	42582272	40405104	57
KPN174	JAJNWL000000000	299	Blood	46681268	44560124	56
KPN175	JAJNWK000000000	432	Blood	47911570	45831408	57
KPN176	JAJNWJ000000000	4998	Blood	48286316	46263410	56
KPN178	JAJQLR000000000	412	Blood	41845910	39942568	57
KPN179	JAJNWI000000000	375	Blood	34706256	33097602	56
KPN180	JAJQLQ000000000	11	Blood	45993464	43915550	57
KPN181	JAJNWH000000000	3640	Blood	43770326	41555246	57
KPN182	JAJNWG000000000	35	Blood	39525880	37587986	57
KPN183	JAJNWF000000000	45	Blood	38480166	36624716	57
KPN184	JAJNWE000000000	15	Blood	37069962	35292398	57
KPN185	JAJNWD000000000	290	Blood	43029526	41074800	56
KPN186	JAJNWC000000000	11	Blood	36703570	35095924	57
KPN187	JAJNWB000000000	985	Blood	40674176	38709916	57
KPN188	JAJNWA000000000	5889	Blood	38983198	37020456	56
KPN189	JAJNVZ000000000	11	Blood	41397102	39542546	57
KPN190	JAJNVY000000000	48	Blood	37522174	35784302	57

Table S4 Recombination events of ST11-KL64 CRKP strains.

Recombinant Detection	Start	End	nSNPs	Length(bp)
RD1	68395	72307	33	3912
RD11	98313	99039	15	726
RD12	126963	127860	11	897
RD13	142889	144216	15	1327
RD14	248647	250772	12	2125
RD2	302677	309280	27	6603
RD15	333596	335728	15	2132
RD16	384888	385384	6	496
RD17	495036	496860	17	1824
RD3	658410	669868	18	11458
RD18	700758	701961	28	1203
RD19	802121	802909	17	788
RD20	1140379	1141762	69	1383
RD4	1674111	1681068	62	6957
RD21	1805498	1806536	6	1038
RD5	1942028	1949004	74	6976
RD22	1999516	1999957	67	441
RD6	2320117	2328726	68	8609
RD23	2365569	2367411	26	1842
RD24	2396544	2399076	12	2532
RD25	2506479	2507730	13	1251
RD7	2727813	2732085	9	4272
RD26	2919789	2920341	6	552
RD27	3068517	3068772	6	255
RD8	3152777	3156186	8	3409
RD28	3246496	3247156	6	660
RD9	3374104	3379171	48	5067
RD29	3543864	3544371	5	507
RD30	3551046	3551682	10	636
RD31	3660759	3662949	37	2190
RD32	3726527	3727464	24	937
RD33	3753434	3754818	30	1384
RD34	3772491	3772915	5	424
RD10	3908358	3916598	54	8240
RD35	3976035	3976750	10	715

Table S5 Genomic information of 122 ST11-KL64 CRKP strains download from the Pathosystems Resource Integration Center (PATRIC).

Strain Name	wzi	K_locus	Bla_Carb_acquired	ST	Country	Collection Year
573.14875	wzi64	KL64	KPC-2	ST11	China	2016
573.14877	wzi64	KL64	KPC-2	ST11	China	2016
573.14885	wzi64	KL64	KPC-2	ST11	China	2017
573.14887	wzi64	KL64	KPC-2	ST11	China	2017
573.14892	wzi64	KL64	KPC-2	ST11	China	2017
573.14893	wzi64	KL64	KPC-2	ST11	China	2016
573.14895	wzi64	KL64	KPC-2	ST11	China	2017
573.14900	wzi64	KL64	KPC-2	ST11	China	2016
573.14903	wzi64	KL64	KPC-2	ST11	China	2016
573.14904	wzi64	KL64	KPC-2	ST11	China	2016
573.14905	wzi64	KL64	KPC-2	ST11	China	2016
573.14906	wzi64	KL64	KPC-2	ST11	China	2016
573.14908	wzi64	KL64	KPC-2	ST11	China	2016
573.14909	wzi64	KL64	KPC-2	ST11	China	2016
573.14910	wzi64	KL64	KPC-2	ST11	China	2016
573.14912	wzi64	KL64	KPC-2	ST11	China	2016
573.14915	wzi64	KL64	KPC-2	ST11	China	2016
573.14918	wzi64	KL64	KPC-2	ST11	China	2016

573.14919	wzi64	KL64	KPC-2	ST11	China	2016
573.14920	wzi64	KL64	KPC-2	ST11	China	2016
573.14921	wzi64	KL64	KPC-2	ST11	China	2016
573.14922	wzi64	KL64	KPC-2	ST11	China	2016
573.14925	wzi64	KL64	KPC-2	ST11	China	2016
573.14928	wzi64	KL64	KPC-2	ST11	China	2016
573.14930	wzi64	KL64	KPC-2	ST11	China	2016
573.14931	wzi64	KL64	KPC-2	ST11	China	2016
573.14933	wzi64	KL64	KPC-2	ST11	China	2016
573.14934	wzi64	KL64	KPC-2	ST11	China	2016
573.14935	wzi64	KL64	KPC-2	ST11	China	2016
573.14941	wzi64	KL64	OXA-181	ST11	China	2016
573.14942	wzi64	KL64	KPC-2	ST11	Brazil	2016
573.14943	wzi64	KL64	KPC-2	ST11	Brazil	2016
573.14944	wzi64	KL64	KPC-2	ST11	Milan	2017
573.14947	wzi64	KL64	KPC-2	ST11	China	2016
573.14950	wzi64	KL64	KPC-2	ST11	China	2016
573.14951	wzi64	KL64	KPC-2	ST11	China	2016
573.15323	wzi64	KL64	KPC-2	ST11	Togo	2016
573.15325	wzi64	KL64	KPC-2	ST11	Malaysia	2016
573.15328	wzi64	KL64	KPC-2	ST11	China	2017

573.18689	wzi64	KL64	KPC-2	ST11	China	2019
573.18690	wzi64	KL64	KPC-2	ST11	China	2019
573.18692	wzi64	KL64	KPC-2	ST11	China	2018
573.19854	wzi64	KL64	KPC-2	ST11	China	2019
573.19855	wzi64	KL64	KPC-2	ST11	China	2019
573.19856	wzi64	KL64	KPC-2	ST11	China	2019
573.19881	wzi64	KL64	KPC-2	ST11	China	2018
573.19978	wzi64	KL64	KPC-2	ST11	China	2019
573.22577	wzi64	KL64	KPC-2	ST11	China	2018
573.25802	wzi64	KL64	KPC-2	ST11	Brazil	2018
573.25809	wzi64	KL64	KPC-2; OXA-48	ST11	Bulgaria	2018
573.26005	wzi64	KL64	KPC-2	ST11	China	2017
573.26023	wzi64	KL64	KPC-2	ST11	South Africa	2020
573.26024	wzi64	KL64	KPC-2	ST11	Brazil	2020
573.26025	wzi64	KL64	KPC-2	ST11	Brazil	2020
573.26026	wzi64	KL64	KPC-2	ST11	Brazil	2020
573.26027	wzi64	KL64	KPC-2	ST11	Brazil	2020
573.26029	wzi64	KL64	KPC-2	ST11	Brazil	2019
573.27273	wzi64	KL64	KPC-2	ST11	China	2018
573.27274	wzi64	KL64	KPC-2	ST11	China	2018
573.27275	wzi64	KL64	KPC-2	ST11	China	2018

573.27286	wzi64	KL64	KPC-2	ST11	China	2019
573.27287	wzi64	KL64	KPC-2	ST11	China	2019
573.27288	wzi64	KL64	KPC-2	ST11	Thailand	2018
573.27289	wzi64	KL64	KPC-2	ST11	Thailand	2018
573.27290	wzi64	KL64	KPC-2	ST11	Thailand	2018
573.27291	wzi64	KL64	KPC-2	ST11	Thailand	2018
573.27292	wzi64	KL64	KPC-2	ST11	USA	2016
573.27293	wzi64	KL64	KPC-2	ST11	USA	2016
573.27295	wzi64	KL64	KPC-2	ST11	Nigeria	2015
573.27978	wzi64	KL64	KPC-2	ST11	China	2014
573.27979	wzi64	KL64	KPC-2	ST11	China	2015
573.27980	wzi64	KL64	KPC-2	ST11	China	2015
573.27982	wzi64	KL64	KPC-2	ST11	China	2015
573.27983	wzi64	KL64	KPC-2	ST11	China	2015
573.27984	wzi64	KL64	KPC-2	ST11	China	2015
573.27985	wzi64	KL64	KPC-2	ST11	China	2015
573.27987	wzi64	KL64	KPC-2	ST11	China	2015
573.27989	wzi64	KL64	KPC-2	ST11	China	2015
573.27990	wzi64	KL64	KPC-2	ST11	China	2014
573.29375	wzi64	KL64	KPC-2	ST11	China	2015
573.29378	wzi64	KL64	KPC-2	ST11	China	2015

573.29379	wzi64	KL64	KPC-2	ST11	China	2015
573.29387	wzi64	KL64	KPC-2	ST11	China	2014
573.29388	wzi64	KL64	KPC-2	ST11	Greece	2014
573.29389	wzi64	KL64	KPC-2	ST11	Greece	2014
573.29391	wzi64	KL64	KPC-2	ST11	Greece	2013
573.29392	wzi64	KL64	KPC-2	ST11	Greece	2013
573.30920	wzi64	KL64	KPC-2	ST11	USA	2013
573.30922	wzi64	KL64	KPC-2	ST11	USA	2013
573.30924	wzi64	KL64	KPC-2	ST11	USA	2013
573.30925	wzi64	KL64	OXA-181	ST11	USA	2014
573.30927	wzi64	KL64	KPC-2	ST11	USA	2015
573.30931	wzi64	KL64	KPC-2	ST11	China	2014
573.30935	wzi64	KL64	KPC-2	ST11	China	2013
573.30939	wzi64	KL64	KPC-2	ST11	China	2014
573.31037	wzi64	KL64	KPC-2	ST11	USA	2014
573.31039	wzi64	KL64	KPC-2	ST11	USA	2014
573.31041	wzi64	KL64	KPC-2	ST11	India	2014
573.31587	wzi64	KL64	KPC-2	ST11	China	2015
573.31589	wzi64	KL64	KPC-2	ST11	China	2015
573.32428	wzi64	KL64	KPC-2	ST11	China	2015
573.32429	wzi64	KL64	KPC-2	ST11	China	2015

573.32517	wzi64	KL64	KPC-2	ST11	China	2015
573.33820	wzi64	KL64	KPC-30	ST11	China	2015
573.35015	wzi64	KL64	KPC-2	ST11	China	2015
573.35069	wzi64	KL64	KPC-2	ST11	Spain	2013
573.35085	wzi64	KL64	KPC-2	ST11	China	2012
573.35086	wzi64	KL64	KPC-2	ST11	China	2012
573.35088	wzi64	KL64	KPC-2	ST11	China	2012
573.35651	wzi64	KL64	KPC-2	ST11	China	2012
573.35653	wzi64	KL64	KPC-2	ST11	China	2013
573.35654	wzi64	KL64	KPC-2	ST11	China	2013
573.35655	wzi64	KL64	KPC-2	ST11	China	2013
573.35656	wzi64	KL64	KPC-2	ST11	China	2013
573.35665	wzi64	KL64	KPC-2	ST11	Nigeria	2015
573.35669	wzi64	KL64	KPC-2	ST11	Brazil	2015
573.35670	wzi64	KL64	KPC-2	ST11	Brazil	2015
573.35671	wzi64	KL64	KPC-2	ST11	Brazil	2015
573.35677	wzi64	KL64	KPC-2	ST11	Brazil	2015
573.35682	wzi64	KL64	KPC-2	ST11	Brazil	2014
573.35685	wzi64	KL64	KPC-2	ST11	Brazil	2014
573.35686	wzi64	KL64	KPC-2	ST11	Spain	2012

Figures

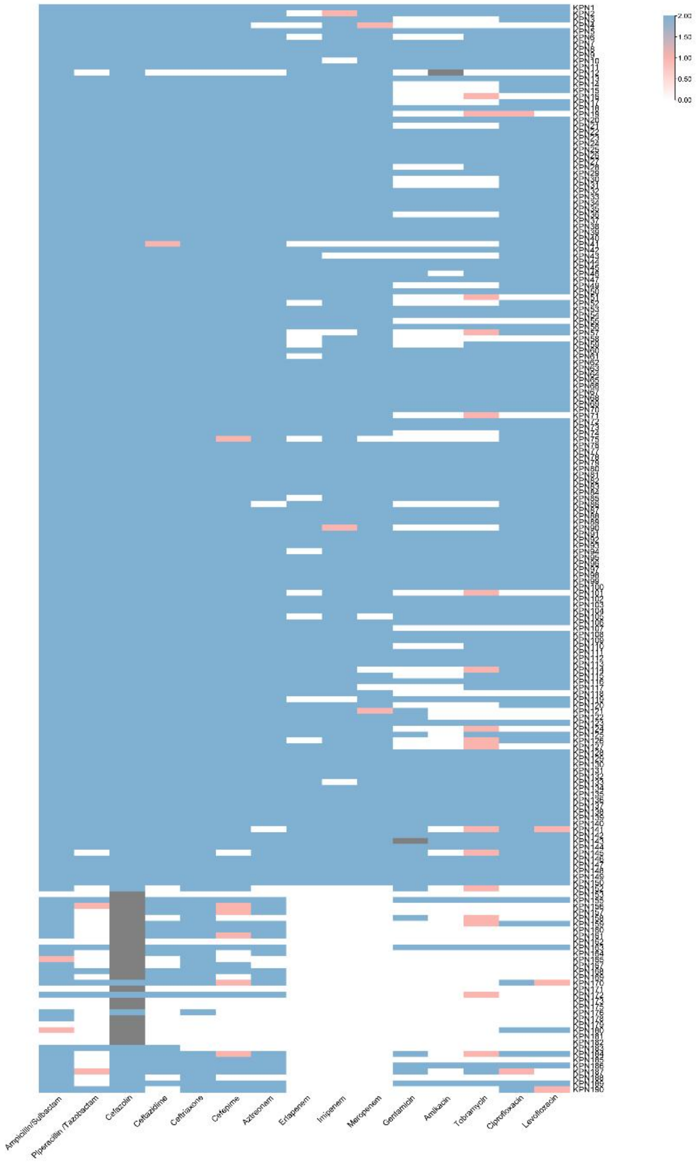

Figure S1 Antibiotic resistance heatmap. The heatmap was constructed by TBtools. The blue square represents drug-resistant, pink square means intermediate results, white square means drug-susceptible, and grey square means missing data. These 185 *Klebsiella pneumoniae* isolates causing bloodstream infection showed high resistance to cephalosporins (100%), aminoglycosides (89.7%) and quinolones (89.7%).

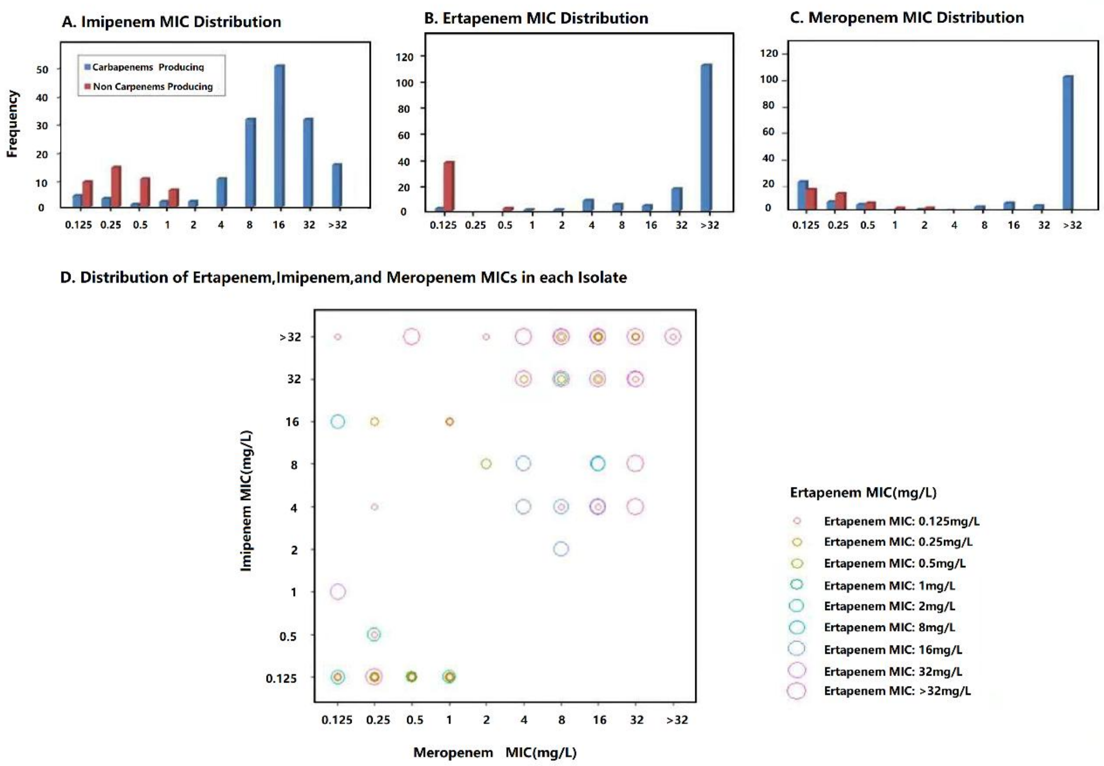

Figure S2 Antibiotic susceptibility results of 185 strains to three carbapenem drugs. The histogram shows the MICs distribution. The bubble chart reflects the relationship between MICs of the three antibiotics, the bigger bubble means that the MICs are larger. The MICs of imipenem were more evenly distributed at different values when compared with ertapenem and meropenem.

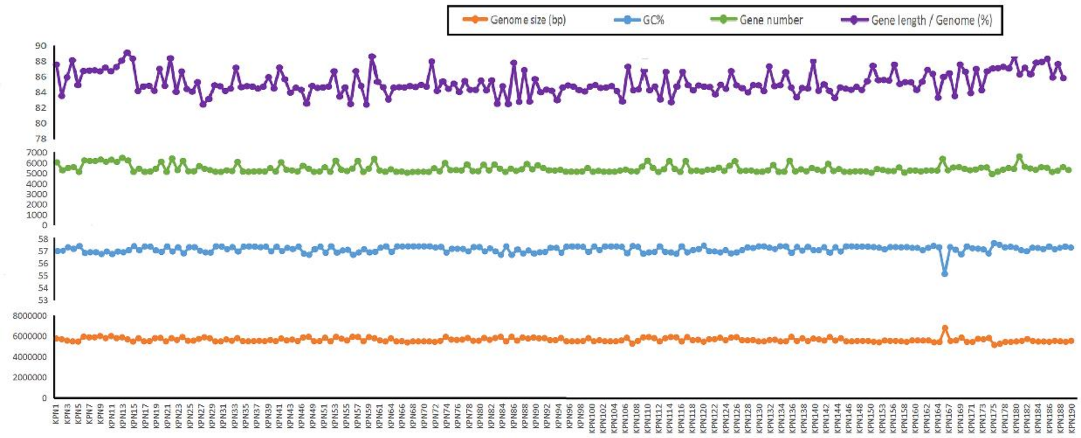

Figure S3 Assembly of 185 *Klebsiella pneumoniae* isolates. The line chart represents the assembly information of 185 isolates. Each dot represents an isolate and the line of purple, green, blue and orange mean gene length, gene number, GC% and genome size, respectively.

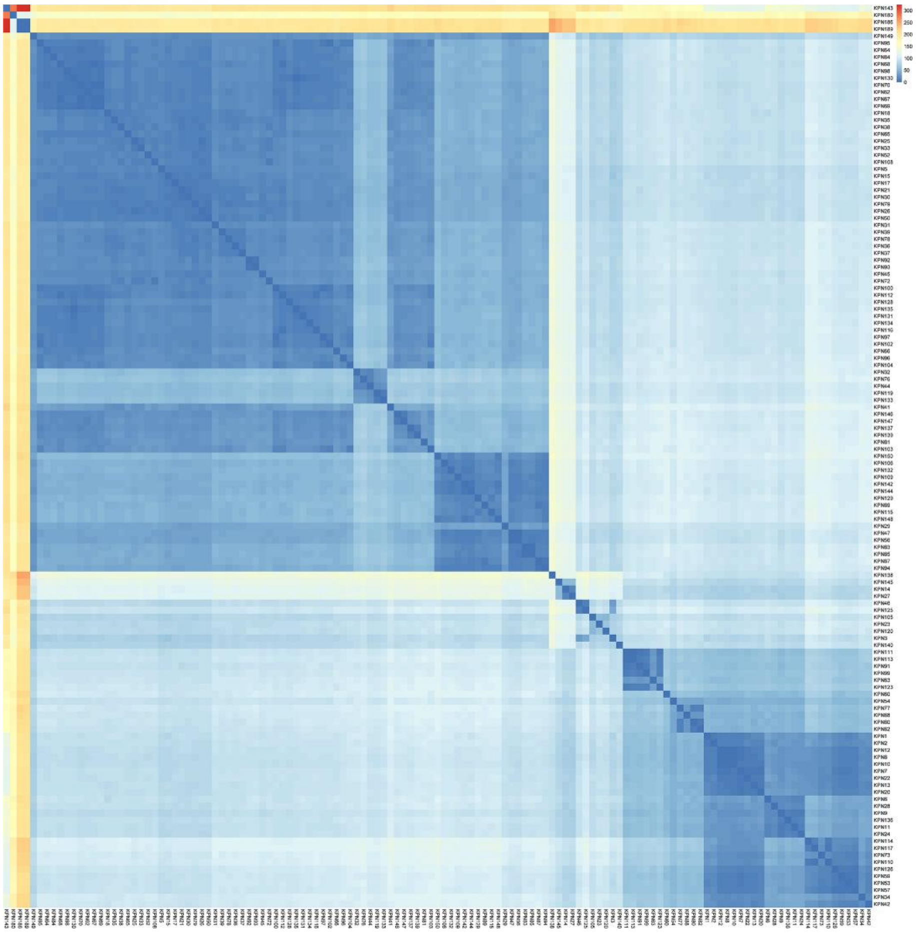

Figure S4 SNP matrix plot of 129 ST11 *Klebsiella pneumoniae* isolates. The closer the differentiation distance between the isolates, the greater the possibility of transmission. the dark blue color in the figure indicates that there is an evolutionary relationship of transmission of the strains.

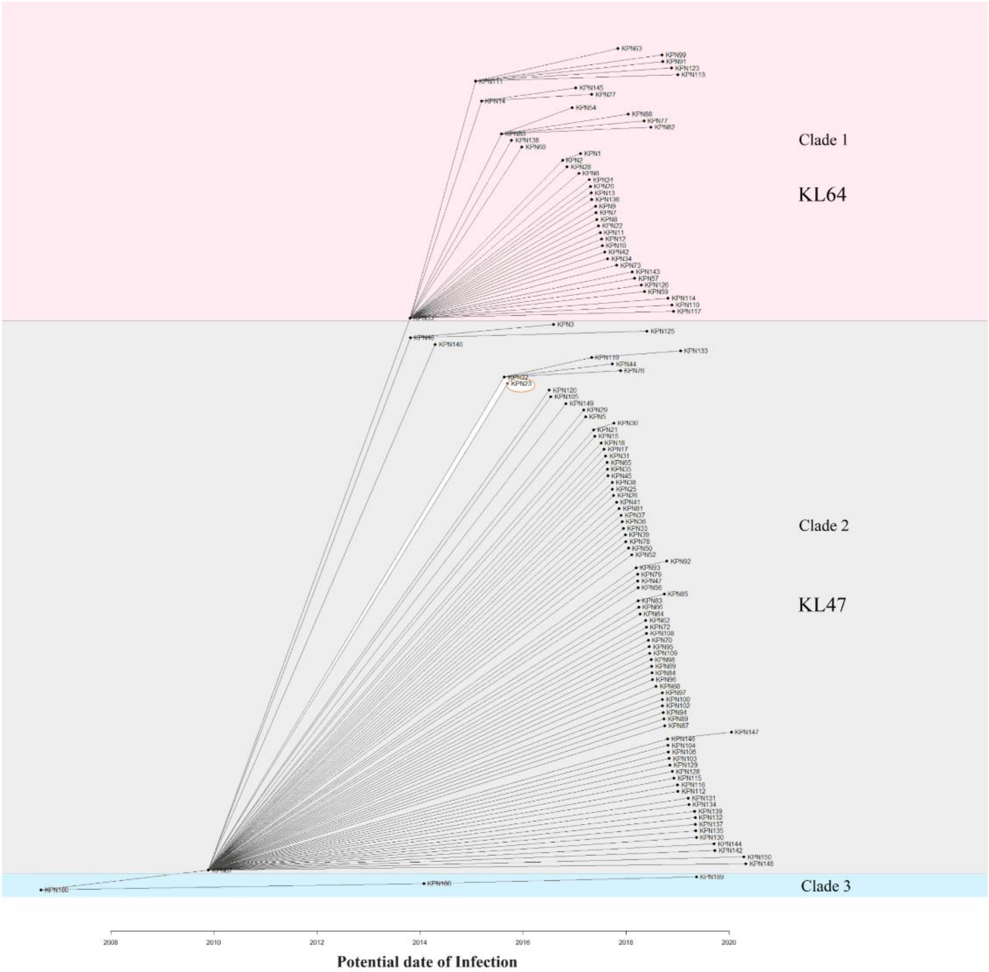

Figure S5 Transmission and evolution tree of 129 ST11 *Klebsiella pneumoniae* isolates. The x-axis provides the potential date of infection, and the y-axis has no means. Filled dots represent sampled isolates and unfilled dots represent un-sampled isolates, by which transmission and outbreak events were inferred. Pink, grey and blue area represent Clade 1, Clade 2 and Clade 3, respectively. Strains in Clade 1 belonged to ST11-KL64. And strains in Clade 2 all belonged to KL47 except for KPN23, which was marked with a circle, for having no clear serotype. Clade 3 included 3 ST11 CSKP strains, it showed that serotype evolved from KL15 to KL105.

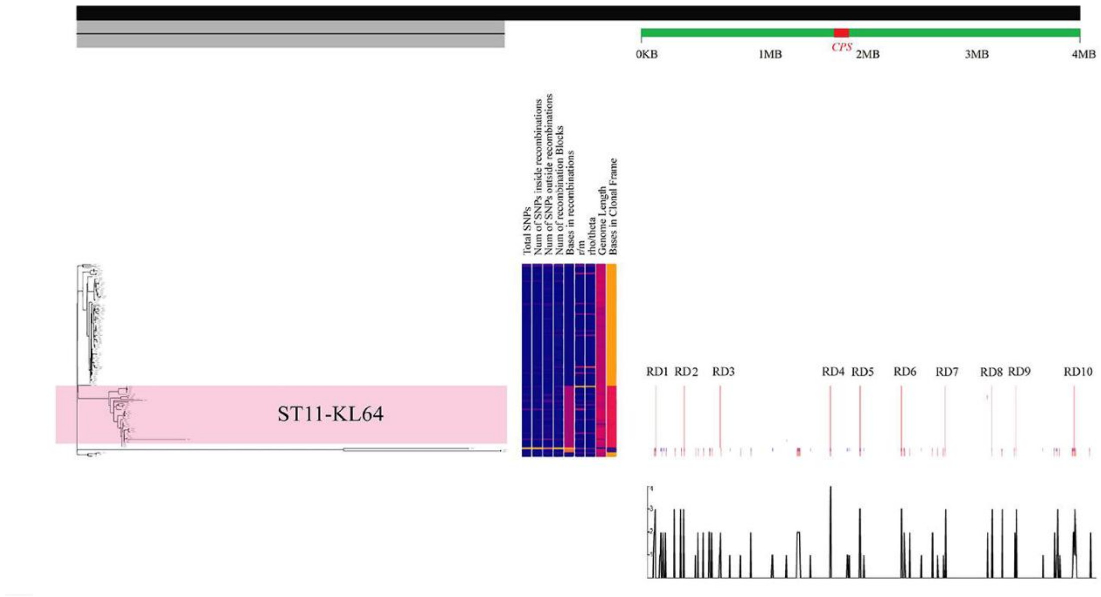

Figure S6 Screenshot of recombination events inferred in a collection of ST11 *Klebsiella pneumoniae* genomes by Gubbins and figure produced with Phandango. Ten recombinant genomic regions > 3 kb (RD1-RD10) were predicted by Gubbins in all ST11-KL64 CRKP isolates highlighted in pink, and two of these were localized around the *cps* region. Very small recombinant regions cannot be seen in this figure but the details of all recombinant regions are listed in Supplemental Table S4.

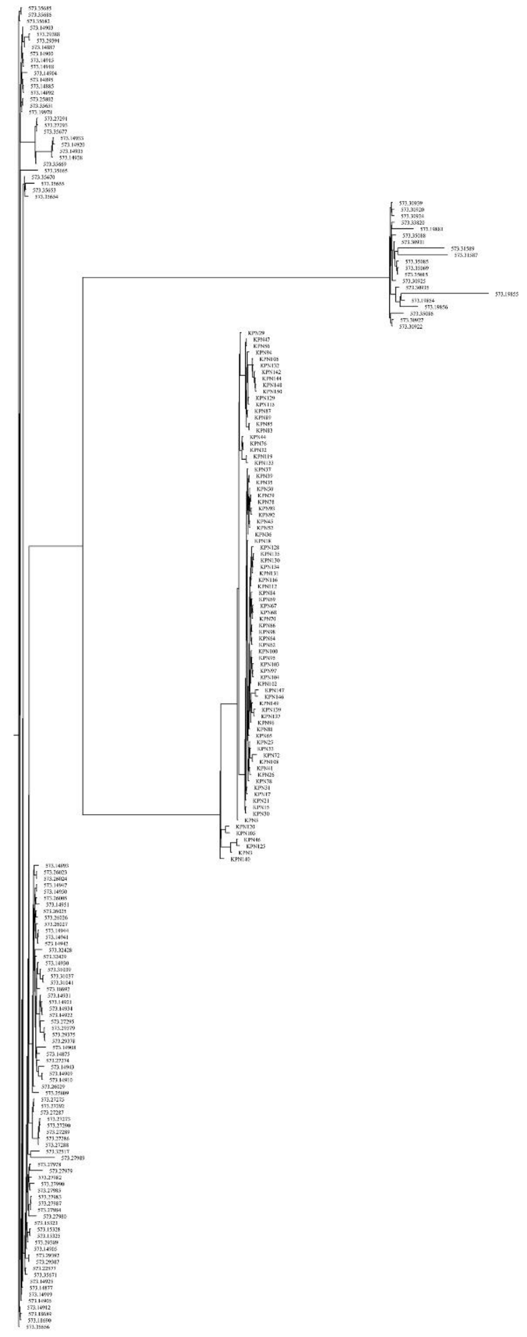

Figure S7 Phylogenetic analysis of 122 ST11-KL64 CRKP downloaded from the Pathosystems Resource Integration Center (PATRIC) database and 82 ST11-KL47 CRKP from our collection. The core genome was constructed using Roary v3.13.0, recombination was removed using ClonalFrameML v1.12 and the phylogenetic tree was drawn using the maximum likelihood method. 122 strains from the public database are shown in Supplemental Table S5.

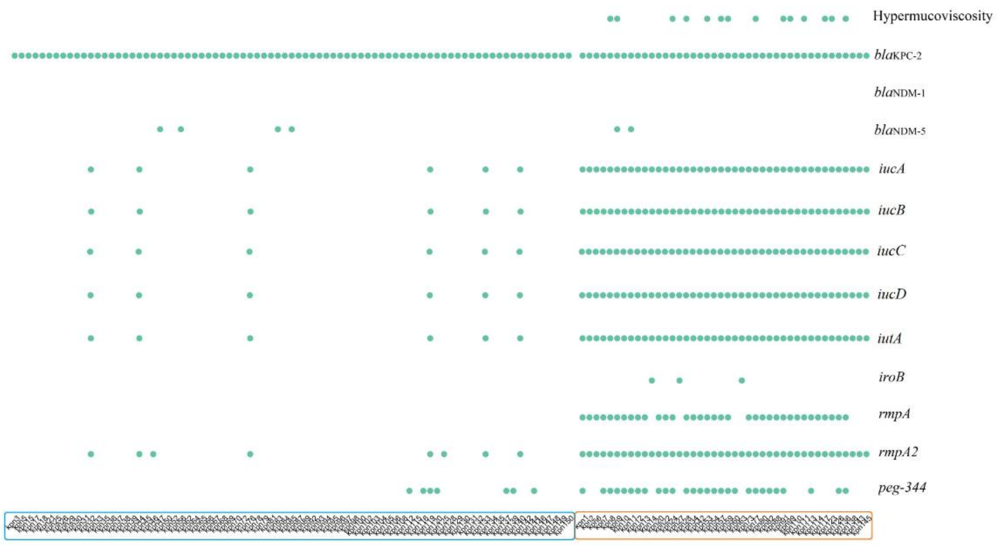

Figure S8 Virulence and carbapenemase encoding genes carried by ST11-KL47 and ST11-KL64. The green spot means that the corresponding gene is present in the strain. The strains in the blue box belonged to ST11-KL47, and the strains in the orange box belonged to ST11-KL64. More virulence genes such as *rmpA2*, *iucABCD* and *iutA* were detected in ST11-KL64 strains. And hypermucovisity phenotype only appeared in ST11-KL64 strains.

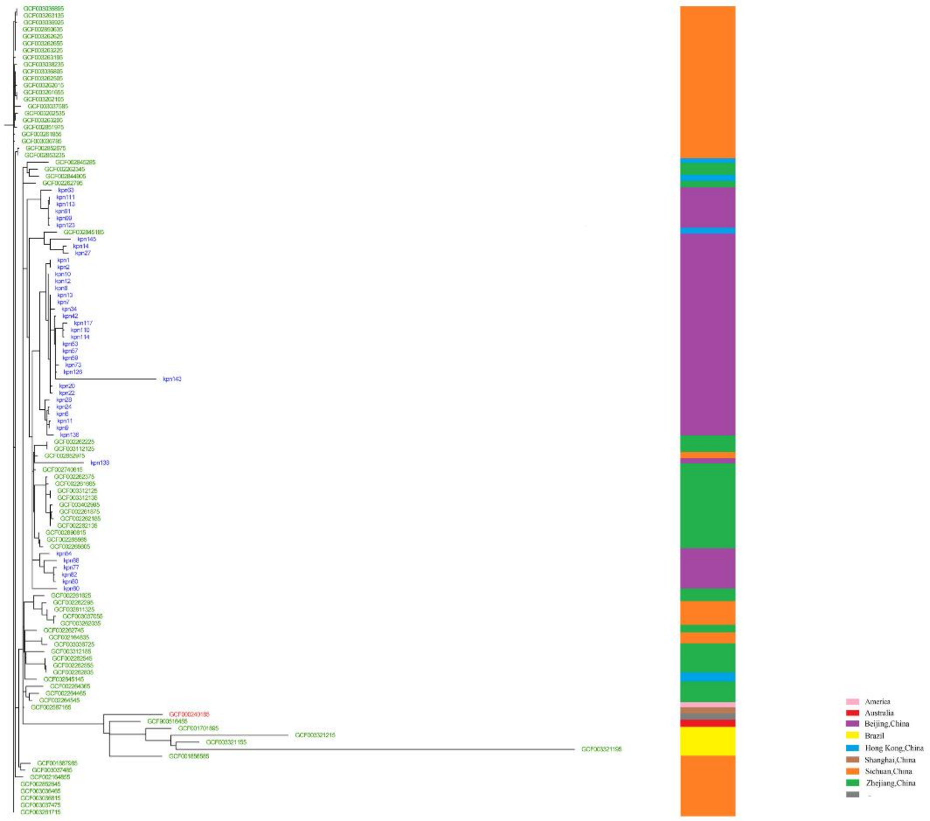

Figure S9 Phylogenetic analysis of 115 ST11-KL64 *Klebsiella pneumoniae* isolates from our study and public database. The phylogenetic tree was acquired by mapping all sequence reads to the hybrid assembly of GCF_000240185 and removing the recombined regions from the alignment, then constructed by snippy v3.1. 83 ST11-KL64 isolates with green font were sequenced in previous studies and downloaded from GenBank. And last 42 ST11-KL64 isolates with blue font were collected from a tertiary hospital during 2017-2020 in this study. Different colored squares represent the corresponding city or country.

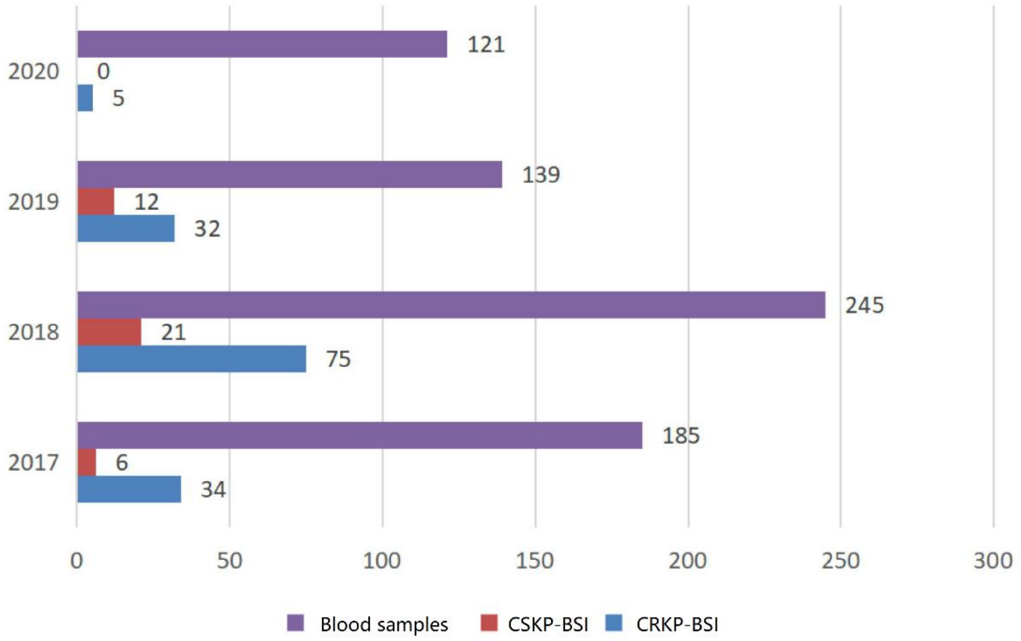

Figure S10 The number of blood samples in total and the number of carbapenem-resistant or carbapenem-susceptible isolates used in this study. The histogram shows the total number of blood samples collected each year from 2017-2020, as well as the number of CRKP and CSKP isolates resulting in bloodstream infections.
